# Deciphering Z-scheme Charge Transfer Dynamics in Heterostructure NiFe-LDH/N-rGO/g-C_3_N_4_ Nanocomposite for Photocatalytic Pollutant Removal and Water Splitting Reactions

**DOI:** 10.1038/s41598-019-39009-4

**Published:** 2019-02-21

**Authors:** Susanginee Nayak, K. M. Parida

**Affiliations:** Centre for Nano Science and Nano Technology, Siksha ‘O’ Anusandhan Deemed to be University, Bhubaneswar, 751030 Odisha India

## Abstract

A series of heterostructure NiFe LDH/N-rGO/g-C_3_N_4_ nanocomposite were fabricated by combining calcinations-electrostatic self-assembly and hydrothermal steps. In this method, negatively charged N-rGO was electrostaticaly bonded to the self-assembled interface of n-n type g-C_3_N_4_/NiFe LDH hybrid. XRD and AFM results revealed successful formation of heterostructure nanocomposite due to the coupling effect of exfoliated NiFe LDH nanosheets with N-rGO and g-C_3_N_4_. Among the as synthesized heterostructure, CNNG3LDH performed superior photocatalytic activities towards 95 and 72% mineralization of RhB and phenol. Furthermore, CNNG3LDH could achieve the highest photocatalytic H_2_ evolution rate of 2508 μmolg^−1^2h^−1^ and O_2_ evolution rate of 1280 μmolg^−1^2h^−1^ under visible light irradiation. The CNNG3LDH possess lowest PL intensity, reduced arc of the Nyquist plot (43.8 Ώ) and highest photocurrent density (−0.97 mA cm^−2^) which revealed effective charge separation for superior photocatalytic activities. TRPL spectral results reveal the synergistic effect of layered component in CNNG3LDH for achievable higher life time of excitons of ~16.52 ns. In addition, N-rGO mediator based Z-scheme charge transfer mechanisms in CNNG3LDH were verified by the ESR and TA-PL studies. Enriched oxygen vacancy type defects in NiFe LDH and N-rGO mediated Z-scheme charge transfer mechanistic path strongly manifest the superior photocatalytic activities of the heterostructure materials.

## Introduction

With the rapid progression of modern civilization, environmental pollution and energy crises have become the focus of worldwide apprehension. Nevertheless, a sign of relieve is perceived when the scientists from all over the globe come up with a promising solution known as semiconductor photocatalysis^1^. In this scenario, photocatalysis has emerged as green technology for the solution of energy crises and deteriorating environmental chaotic situation. Hence, expedition of highly efficient and low cost catalyst to accelerate photocatalysis processes is highly urged for making greener earth^2^. Amongst the bimetallic layered double hydroxide (LDH) based on 3*d* transition-metals, NiFe LDH have attracted increasing attention due to their abundant resource, low cost, non-toxicity, rich redox activity, and eco-friendliness accompanied by relatively narrow band gap for excellent visible light-harvestation ability^3–5^. However, irrespective of all these advantages, NiFe LDHs exhibit high electrical resistance and irreversible aggregation of their exfoliated nanosheets that questions the stability in nanometric region followed by precaution needed during the preparation methods owing to large differences in solubility product between the two metal moieties (Ni(OH)_2_, Ksp = 5.5 × 10^−16^ and Fe(OH)_3_, Ksp = 2.8 × 10^−39^). Therefore, some vital research works have been carried out by various scientific groups to overcome all these lacunas of NiFe LDH. Yet, all these above difficulties of NiFe LDH could be practically overcome by the formation of heterostructure material with the synergistic effect of constituent semiconductor. In addition heterostructure formation of NiFe LDH causes exfoliation of bulk NiFe LDH to NiFe LDH nanosheets and the edge-sharing octahedral MO_6_ layers could creates surface defects through oxygen vacancies, which are responsible for the formation of coordinatively unsaturated metal centers for the harvestation of sunlight by modification of electronic structure^6^. Furthermore, a number of reports are available highlighting work on NiFe LDH based heterostructure material for significant catalytic activity as in TiO_2_/graphene/NiFe LDH^6^, carbon quantum dot/NiFe LDH^7^, Cu nanowire shelled with NiFe LDH^8^, MoO_4_^2−^/NiFe LDH^9^, graphene/NiFe LDH^10^, FeOOH/NiFe LDH^11^, NiCoO/NiFe LDH^12^, and NiCo_2_S_4_/NiFe LDH^13^. Nevertheless, there are rare discussions on fabrication and stabilization effect of NiFe LDH nanosheets for hybrid photocatalytic system. Inspired by the hard-core chemistry behind the structural defects of exfoliated NiFe LDH, we are motivated a lot for the fabrication of NiFe LDH based heterostructure material to enhance the photocatalytic activities by utilizing the advantages of the strong coupling effects of the constituent semiconductor component.

Accelerating surface reaction kinetics and anti-recombination of charge pairs are important characteristic of heterostructure photocatalyst. Therefore, metal-free polymeric layered graphitic carbon nitride (g-C_3_N_4_) with 2D structure similar with graphene consisting of earth-abundant elements of carbon and nitrogen has been recognized as novel photocatalyst for the integration of NiFe LDH owing to their smooth carrier charge mobility and catalytic activities^14^. Recent literature reports also reveals that coupling of g-C_3_N_4_ with other layered structure semiconductor material is an effective approach for achieving targeted photocatalytic performances^15–18^. By our earlier work, n-n type g-C_3_N_4_/NiFe LDH hybrid nanocomposite have been proven to be highly efficient energy materials for visible light driven water oxidation and reduction reactions^5^. However, stabilization and dispersion of transition metals of exfoliated LDH nanosheets on g-C_3_N_4_ matrix could be major challenge due to the strong irreversible agglomerates of LDH nanosheets and requires higher surface area for effective results in photocatalysis as in ZnCr LDH/GO^19^.

N-doped graphene matrix was proven to be more efficient for anchoring metal atoms (clusters) of 2D nanolayers^20,21^. The doping of nitrogen nonmetal over the networks of graphene surface is capable enough to facilitate charge transfer across the adjacent carbon atoms and thereafter augmented the photocatalytic activity of graphene-related materials^22^. The introduction of N-doped graphene into the heterostructure assembly of g-C_3_N_4_/NiFe LDH hybrid behaves as an electronic mediator that could stronger the interfacial interactions charge transfer due to surface area and conductivity benefits. The layered heterostructure between exfoliated NiFe LDH nanosheet, N-doped graphene and g-C_3_N_4_ could decrease the charge diffusion transport time and path in comparison with their pristine counter one. Strong and stable interfacial contact is the root cause for enhancement in catalytic performance of heterostructure nanomaterials. What is more, N-doped graphene framework structure have greater advantages than 2D graphene framework and provide more efficient surface area *via* strong π– π interaction to directly couple transition metal atoms sites of exfoliated NiFe LDH nanosheets, leading to fast electron transfer kinetics and excellent stability^23–26^. Authors group have also reported various heterostructure based materials such as CeO_2_/MgAl-LDH, Co(OH)_2_/ZnCr LDH, Ag@Ag_3_PO_4_/g-C_3_N_4_/NiFe LDH, MgO/MgCr_2_O_4_ derived from MgCr-LDH nanosheets for photocatalytic H_2_ and O_2_ production with Cr(VI) reduction and phenol degradation activities^27–30^. Among the above promising results of NiFe LDH based heterostructure materials^6–13^, there have been no reports on controlled coupling of NiFe LDH and g-C_3_N_4_ with 2D N-doped graphene frameworks structure for photocatalytic removal of organic pollutant with productions of clean energy of H_2_ and O_2_.

Herein, we report the fabrication of heterostructure material comprising of exfoliated NiFe LDH nanosheets, N-doped graphene (N-rGO) and g-C_3_N_4_. At first, N-GO/g-C_3_N_4_ hybrid was synthesized by heat treatment at 550 °C using melamine as precursor for N-doping into the graphene lattice as well as forming g-C_3_N_4_. The above N-GO/g-C_3_N_4_ hybrids were dispersed in deionized water and further immersed into the NiFe LDH nanosheets suspension in formamide and afterwards the whole mixture was transferred into a Teflon lined autoclave for hydrothermal treatment. Under hydrothermal treatment, the N-GO was reduced to N-rGO and simultaneously the electron enriched C-N bonds present in negatively charged N-rGO and negatively charged 2D layered g-C_3_N_4_, electrostaticaly anchored with the positively charged NiFe LDH nanosheets forming heterostructure NiFe LDH/N-rGO/g-C_3_N_4_ nanocomposite. The separation and transfer of charge carrier path across the interface of the heterostructure NiFe LDH/N-rGO/g-C_3_N_4_ was proposed by tallying both the experimental and characterization technique results in which N-rGO solid state mediator based Z-scheme mechanism was established for the first time^31–33^. Lastly, the remarkable photocatalytic performances of the heterostructure towards mineralization of stable organic pollutants (Rhodamine B and Phenol) and visible light driven H_2_ and O_2_ productions was attributed to the outstanding synergistic coupling effects of (i) effective charge carrier migration and separation promoted by oxygen vacancies of exfoliated NiFe LDH nanosheets and further mediated by N-doped rGO, (ii) oxidation ability arising from NiFe LDH and (iii) superior reduction reaction activity due to g-C_3_N_4_.

## Results and Discussions

### Structural and surface charge analysis

**Figure 1:**
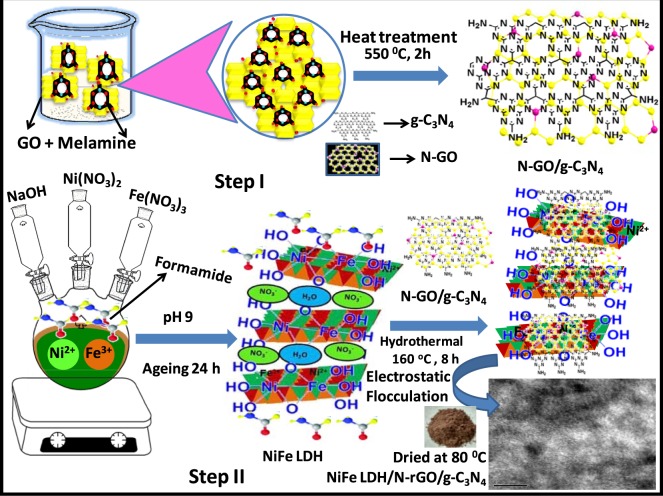
Synthetic steps of heterostructure CNNGxLDH.

**Figure 2:**
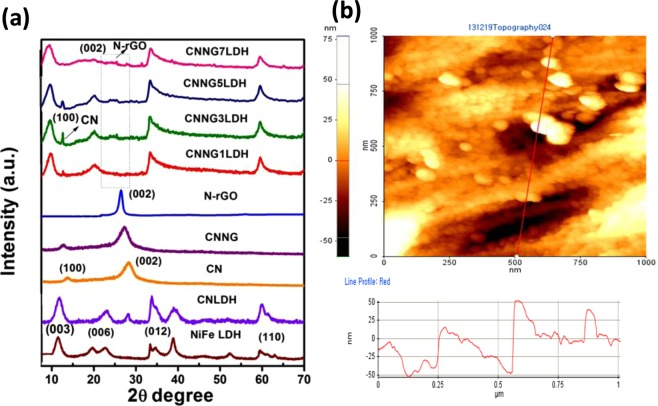
(**a**) XRD pattern of NiFe LDH, CN, CNLDH, N-rGO, CNNG and CNNGxLDH, and (**b**) AFM image of CNNG3LDH.

LDH layer charge density plays an important role during its exfoliation processes owing to the isomorphous substitution of M^2+^ by M^3+^ in their brucite like layered structure^34^. Based on these versatile natures of LDH, the metal cation ratio of 5:1 in NiFe LDH system was found to be smoother for exfoliation process^5^. The schematic representation as shown in Fig. 1 depicts that positively charged NiFe LDH nanosheets could be obtained after exfoliation in formamide solution, which subsequently heteroassembled with negatively charged N-GO/g-C_3_N_4_ (CNNG) *via* electrostatic self-assembly and hydrothermal treatment to generate heterostructure NiFe LDH/N-rGO/g-C_3_N_4_ nanocomposite (CNNGxLDH, x = 1, 3, 5 and 7 wt% of GO). The XRD patterns of the heterostructure CNNGxLDH were analysed thoroughly and compared with pristine NiFe LDH, pristine CN and CNLDH composite material (Fig. 2a). The diffraction pattern of NiFe LDH (NO_3_^−^) consists of symmetrical patterns of (003) and (006) planes and asymmetrical patterns of (015) and (018) planes. The d-spacing value of (003) plane of NiFe LDH is 0.48 nm, which signifies the presence of NO_3_^−^ anions^5^. In contrast, intensity of (003) and (006) characteristic peaks of NiFe LDH was reduced and the position shifted to lower angle after exfoliation and coupling with N-rGO/g-C_3_N_4_ (CNNG), which indicate the complete exfoliation of pristine NiFe LDH (NO_3_^−^) into NiFe LDH nanosheets^35^. The diffraction patterns of pristine CN were well consistent with the characteristic (100) plane at 2θ value of 13^ο^ and (002) plane at 27.5^ο^. The strong diffraction pattern of (002) plane of CN was recognized as the packing of the π-conjugated aromatic cyclic rings^5^. In CNLDH composite, the XRD pattern exhibits characteristic diffraction patterns of both NiFe LDH and CN, while the main characteristic peaks of CN at 2θ value of 27.5^ο^ was prominent without interfering the feature peak of NiFe LDH^5^. However in XRD patterns of heterostructure CNNGxLDH, the characteristic peak intensities of (003) and (006) planes of NiFe LDH were very weak as compared to the pristine NiFe LDH, which indicate that only a small part of LDH nanosheets were reassembled. The peak intensities of CNNG3LDH was significantly lower and broader than those for other series of the heterostructure, implying the better dispersion and interaction of exfoliated NiFe LDH nanosheets with N-rGO matrix. This may be attributed to the heteroatom in N-rGO for effective bonding and dispersion of transition metal atoms of 2D materials^36–38^. Notably the N-rGO peak was found to be at 26.3° as shown in Fig. 2a^39^. Importantly, the (002) plane of N-rGO becomes broad and shifted from 26.3° to 26.1° in CNNG3LDH, which are attributed to the structural changes of carbon and strong chemical coupling during the N doping over GO with simultaneous formation of CN in CNNG hybrid as described in Figs 1, 2a^40^. Additionally, (100) plane of CN was shifted from 13° to 12.8° in heterostructure CNNGxLDH, which strongly support the strong coupling and presence of CN in CNNGxLDH. The (002) plane of CN in CNNG is shifted from 27.5° to 27.1° due to the interference of (002) plane of CN with (002) plane of N-rGO. In this way, XRD pattern confirmed the successful structural coordination of NiFe LDH and CN over N-rGO framework, which resulted in the formation of heterostructure CNNGxLDH nanocomposite.

The atomic force microscopy (AFM) tapping mode was applied to investigate the thickness of the heterostructure CNNG3LDH nanocomposite. The AFM image in Fig. 2b shows that the CNNG3LDH possesses flat and continuous layer of rough surface with defects and some small irregular pores. As shown in Fig. 2b, heterostructure CNNG3LDH with lateral width of 0.25 µm and thickness of 50 nm were found from the cross-sectional analysis. Based on the interlayer distance of (003) plane of NiFe LDH i.e. 0.48 nm from the XRD patterns (Fig. 2a), it is therefore estimated that the CNNG3LDH samples consist of packing of 24 units of nanofold^5^. The higher thickness of CNNG3LDH is due to the presence of some chemisorbed water or organic moieties together with NiFe LDH, N-rGO and CN, which provides sufficient exposure of the active sites for superior photocatalytic activities^10^. The no of units in a nanofold of heterostructure has profound effect on constructing Z-scheme system. Z-scheme type heterostructure photocatalyst is an important system for photocatalytic reactions, where the excited electron of photosystem II (PSII) is transferred into photosystem I (PSI) and recombined with the hole on its surface with the support of electron-transfer mediator^41^. The excited electrons of PSI and the holes of PSII with high reduction and oxidation capability are left and used for the reduction and oxidation reactions, respectively. The broadening of nanofold contact interface provides enough channels for the Z-scheme electron transfer process. However, with further increase in the nanofold results in the thicker 2D component layer, which is less effectively penetrated by incident light to reach the counter 2D component layer surface. Consequently, the thicker 2D component layer extends the distance for the electron transfer from PSII to the PSI surface, which increases the probability of bulk electron-hole recombination^41^. Therefore, an optimal unit of nanofold is suitable for construction of an efficient Z-scheme system.

The functional groups and strong chemical coupling between NiFe LDH, N-rGO and CN in heterostructure CNNG3LDH were further confirmed by FTIR spectra (Fig. S1a in supporting information). The breathing mode of triazine units at 805 cm^−1^ in CNNG3LDH shows clear shifting in comparison to that of CN and CNLDH (808 cm^−1^)^5,29^. The characteristic bands in the region of 1200–1700 cm^−1^ corresponds to the presence of typical stretching modes of C-N and C=N heterocycles for CN, CNLDH, and heterostructure CNNG3LDH. The intensity of O−H absorption band found around 3700–3500 cm^−1^ have largely reduced in CNNG3LDH as compared to pristine NiFe LDH. Such changes could be ascribed to the interaction of NiFe LDH nanosheets with N-rGO and CN resulted in the formation of C-N and C=N bonds, which predominant the O-H absorption band^42^. Notably, N-doping over GO/rGO is further proved by the absorption band at 1639 cm^−1^, which is somewhat partly overlapped with the stretching vibration of C-C and C-N bonds in CNNG3LDH^43^. The bands at around 500–900 cm^−1^ in low frequency region of CNNG3LDH are due to the presence of metal−oxygen and metal-oxygen-metal lattice vibration in brucite layers of NiFe LDH^5,29^.

Raman spectra (Fig. S1b) of heterostructure CNNG3LDH displays the peak at 530 cm^−1^ and are reflections of both Ni-O-Fe and Ni(OH)_2_, which indicates the structural defects of NiFe LDH nanosheets in CNNG3LDH^44,45^. It is also known that the degree of defects in N-rGO could be estimated by the relative intensity ratio of the D to G band (*I*_*D*_*/I*_*G*_). In CNNG3LDH, the D band was found to be at 1353 cm^−1^ and G band was shifted to 1603 cm^−1^ in comparison to GO^46^. The *I*_D_/*I*_G_ peak intensity ratio obtained for G band of CNNG3LDH was 1.01, which denote the reduction of GO to RGO with higher transparency, densities of defects and disorders for stronger coordination of NiFe LDH and CN dispersion. Also, the *I*_*D*_*/I*_*G*_ value of 1.01 in CNNG3LDH suggests that N source has significant contribution on the disorder/defects of rGO. Raman spectroscopy could also be used to investigate the monolayer, bilayer and multilayer characteristics of the graphene and GO materials. The presence of 2D symmetrical peak at 2680 cm^−1^ in CNNG3LDH indicates the complete exfoliation of rGO^47^.

The stability and surface charges of a photocatalyst changes with varying pH and directly influences on their photocatalytic activities. Figure S1c displayed the plot of zeta potential values *vs*. pH for CNLDH and CNNG3LDH, respectively. In CNLDH, negative zeta potential value of −21.33 mV at pH 7 was noticed due to the presence of negatively charged CN over the surface of NiFe LDH^5^. However the dispersed aqueous solution of CNNG3LDH was quite stable in pH range of 5–9 and zeta potential value was found to be −40.4 mV at pH 7. These results reveal the wetting of negatively charged surface of heterostructure due to the introduction of electron rich N-rGO into CNLDH for superior photocatalytic reaction.

### Optical properties analysis

**Figure 3:**
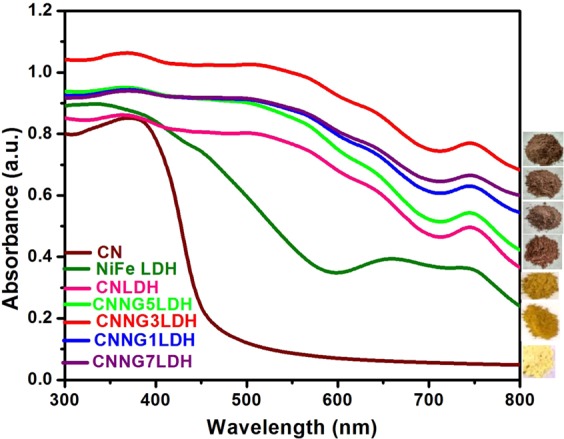
UV–Vis diffuse reflectance spectra of NiFe LDH, CN, CNLDH and CNNGxLDH.

**Figure 4:**
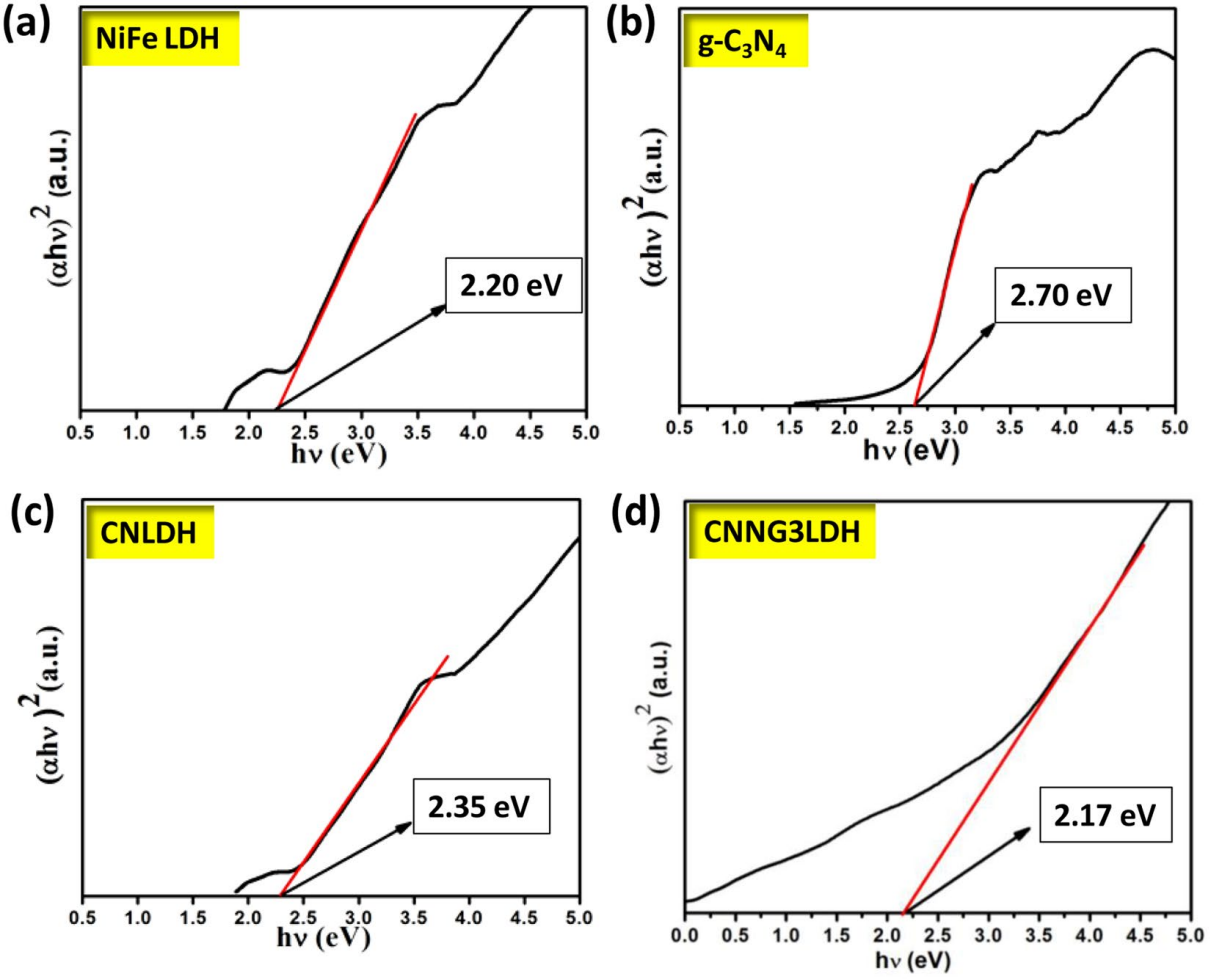
The specific absorption band edges and band gap energy values calculated from UV-Vis DRS of (**a**) NiFe LDH (**b**) CN (**c**) CNLDH and (**d**) CNNG3LDH.

The optical properties of a material are intimately linked with the electronic properties such as band gap energy, charge separation and charge mobility. UV-Vis diffuse reflectance spectra (UV-DRS) in Fig. 3 were thoroughly studied to reveal the optical properties of NiFe LDH, CN, CNLDH and heterostructure CNNGxLDH nanocomposite. In pristine NiFe LDH, the absorption band at 200–400 nm are attributed to the ligand to metal charge transfer spectra (LMCT) between oxygen and metal centre i.e. O2p → Ni/Fe3dt_2g_ orbital in brucite like layer of LDH^5,29^. The absorption band in between 300–800 nm is due to the d-d transition of Ni^2+^ in an octahedral field in NiFe LDH^5,29^. The absorption bands related to spin-allowed transitions of d^8^ configuration of Ni^2+^ ions in an octahedral field are situated at 380 and 740 nm and corresponded to the transition of 3A2g(F) → 3T1g(P) and 3A2g(F) → 3T1g(F), respectively. Likewise, the bands located at 420 and 645 nm corresponded to spin-forbidden transitions 3A2g (F) → 1T2g(D) and 3A2g(F) → 1Eg(D), respectively^29^. The sharp absorption band approximately at 520 nm is due to the transition of Ni^2+^–O–Fe^3+^ to Ni^+^–O–Fe^4+^, which related to the MMCT for oxo-bridged bimetallic linkage^29^. The pristine CN shows an absorption edge at 450 nm with significant absorption in the visible region, which is due to the n–π* transitions of electrons linking the lone pairs on N atoms of the triazine/heptazine heterocyclic ring units. The blue-shift in absorption edge of CNLDH at 441 nm is due to the quantum confinement effect of CN over LDH layered structure with absorption behaviour covering the entire visible spectrum^5,29^. In heterostructure CNNGxLDH, three types of absorption behavior could be detected i.e. LMCT within 200–400 nm, d–d transitions of Ni^2+^ charge transfer in the band region of 400‒712 nm and sharp absorption band at 520 nm is due to the MMCT for oxo-bridged bimetallic linkage of Ni^2+^–O–Fe^3+^ to Ni^+^–O–Fe^4+^ were clearly revealed from the spectra. The dominant nature of NiO_6_ absorption peak in heterostructure is due to the metallic variation of NiO_6_ than FeO_6_ with defects generated during the formation of NiFe LDH nanosheets^48^. The most remarkable finding of the optical studies in heterostructure CNNGxLDH is the red shifting of absorption band from 720–800 nm and the reason may be ascribed in the following points as (i) creation of defects on the surface of NiFe LDH component^48^, (ii) absorption superiority of the N-rGO component^49^, and (iii) quantum confinement with light scattering effects of CN component^50^. However, significant red shifted absorption band with enhanced visible light absorption intensity was detected in CNNG3LDH, which suggest better separation of electron and hole pairs. The band gap energy of semiconductor materials could be calculated by using the following Eq. (1)^48^.

1 $${(\alpha h\nu )}^{1/n}=A(h\nu -{E}_{g})$$

where α is the absorption coefficient, h is the Planck’s constant, ν is the energy of incident light, A is an arbitrary constant, E_g_ is the band gap energy of a semiconductor material. The nature of band gap transition depends upon the n value of a semiconductor, n = 1/2 for direct transition and n = 2 for indirect transition^5,29,30,51^. In this case, LDH and CN both are found to have direct transitions^5,29^. Therefore, the plot of (αhν)^2^ *vs*. hν (Kubelka–Munk function as a function of photon energy) gives the band gap energy value of all the materials by extrapolating the straight line to the hν axis intercept (X-axis) as shown in Fig. 4. The estimated band gap energy value of NiFe LDH, CN, CNLDH and CNNG3LDH was found to be 2.2, 2.7, 2.35 and 2.14 eV, respectively. The band gap energy value of CNLDH was found to be blue shifted than NiFe LDH, which could be attributed to the quantum confinement effect of CN^5,29,50^. The narrowing of band gap value of CNNG3LDH than NiFe LDH, CN and CNLDH clearly evidence the availability of quantum confinement effect, over cluster of nanosheets and defect/vacancies sites^5,25,30^. Though the band gap energy value of individual component i.e. NiFe LDH and CN is quite different but in heterostructure CNNG3LDH exhibit single band gap value, which is due to the strong coupling effect of constituent semiconductor component and simultaneous overlapping of band gap energies due to self assembly of layered to layered structure. Therefore, the band gap energies tuning of the as fabricated heterostructure nanocomposite is due to the accessibility of quantum confinement effect, defect site or oxygen vacancies, which could increase the intensity of light absorption in the visible spectrum for superior photocatalytic activities. The band gap tuning of the materials could be corroborated with the change in color starting from pale yellow to deep yellow and then to reddish brown with the increase in wt% of N-rGO as shown in Fig. 3.

**Figure 5:**
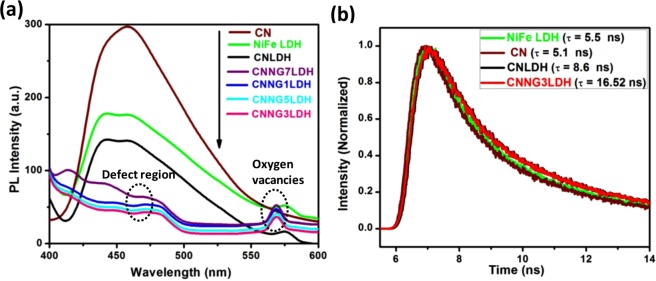
(**a**) Photoluminescence spectra of NiFe LDH, CN, CNLDH and CNNGxLDH measured at excitation energy of 380 nm, (**b**) Time-resolved photoluminescence spectra of NiFe LDH, CN, CNLDH and CNNG3LDH.

The PL emission spectra reveal the extent of charge carrier trapping, transfer and migration of interfacial photogenerated charge pair in a semiconductor material^52,53^. A comparisons of the PL emission spectra of NiFe LDH, CN, CNLDH and heterostructure CNNGxLDH were shown in Fig. 5a. NiFe LDH exhibit three types of emission bands at 442 (strong), 460 (strong) and 575 nm (medium), respectively. The centred peak at 442 and 460 nm were related to the radiative recombination of charge carriers linked to the spin allowed transitions i.e. 3T_2g_ $\to \,$3A_2g_ (F) at 485 and 3T_1g_ (P)$\,\to \,$3A_2g_ (F) at 423 nm for Ni(OH)_2_, respectively^29,54,55^. The stable and continuous emission at 575 nm in NiFe LDH is linked with the surface defects, which serve as trapping site for photoinduced electrons^5,29^. A distinct band-band PL emission of CN was found at 460 nm, which could be approximately equal to the band gap energy value of CN^5,29^. Similarly, CNLDH composite exhibits strong blue shifted emission bands at 450 nm, which corresponds to the quantum confinement of highly conjugated CN layered structure over NiFe LDH for an effective separation of photogenerated charge carriers^5,29^. The heterostructure CNNGxLDH exhibits blue shifted emission peak at ~460 and green shifted emission peak at ~560 nm, respectively. The blue shifted emission band of CNNG3LDH indicated the quantum confinement effect of CN for high light absorption property within all heterostructure for higher rates of interfacial charge transfer efficiency^29^. The intensity of emission peak of CNNG3LDH was significantly reduced than CNLDH and other samples, which was due to the introduction of N-rGO into the self-assembled surface of CNLDH to form a tightly bonded heterostructure nanocomposite for effective carrier charge separation. The green emission band at ~560 nm corresponded to the presence of defects site of oxygen vacancy in CNNG3LDH^29,30^. The new peak in the PL spectra of CNNG7LDH sample at around 410 nm is due to the characteristic spectrum for exfoliated graphene sheet^56^. Due to higher wt% loading of rGO in CNNG7LDH than other samples of the series, the peak is more prominent in CNNG7LDH.The PL peak intensity of green emission band at 568 nm was more intense in heterostructure CNNGxLDH, implying that the energy level of the surface trap oxygen vacancies sites varied due to the transformation of pristine NiFe LDH to NiFe LDH nanosheets^55^. Thus each component of heterostructure plays an important role in the separation and transfer of electron−hole pairs and resulted in the superior visible light photocatalytic activities of heterostructure CNNG3LDH nanocomposite.

Apart from the PL studies, time-resolved photoluminescence spectroscopy gives much idea about the lifetime of excitons and dynamics of photogenerated charge carrier from the resulting decay curves and the data were fitted with a double-exponential function as expressed in Eq. (2)^5,57^.

2 $${\rm{I}}({\rm{t}})={{\rm{A}}}_{1}.{{\rm{e}}}^{(-{\rm{t}}/\tau 1)}+{{\rm{A}}}_{2}.{{\rm{e}}}^{(-{\rm{t}}/\tau 2)}$$

Where τ1 and τ2 are the fluorescent lifetime, A_1_ and A_2_ are the corresponding amplitudes. The best fitted curves with double-exponential function were shown in Fig. 5b. The decay components (τ1 and τ2) and relative amplitudes of the decay species (A1and A2) were summarized in Table S1. The average values of lifetimes emission originated from the overall decay emission time of the samples were calculated by the following Eq. (3)^5^.

3 $$\tau (av)=\frac{A1{\rm{\tau }}{1}^{2}+A2{\rm{\tau }}{2}^{2}}{A1{\rm{\tau }}1+A2{\rm{\tau }}2}$$

The average fluorescence lifetime of CNNG3LDH were calculated to be ${\rm{\tau }}\,$ = 16.52 ns, which is almost double than CNLDH (8.6 ns), and three times or longer than NiFe LDH (5.5 ns), and CN (5.1 ns), respectively. It is also highlighted in our earlier work that layered to layered coupling in CNLDH composite also prolonged the life time of photogenerated charge carrier to 8.6 ns^5^. The prolonged fluorescence lifetime of heterostructure CNNG3LDH nanocomposite was related to the (i) inhibited non-radiative transition after confinement of NiFe LDH and CN over N-rGO^58^, (ii) LMCT transitions in NiFe LDH nanosheets via charge transfer process of O^2−^$\to \,$Ni^2+^, O^2−^$\to \,$Fe^3+^ and oxygen vacancies trap the photoexcited electron from the CB of as fabricated heterostructure nanocomposite and behaves like an intermediate state in the carrier decay process, which were responsible for the observed value of long decay lifetime^5,29,30^.

### Morphological feature analysis

**Figure 6:**
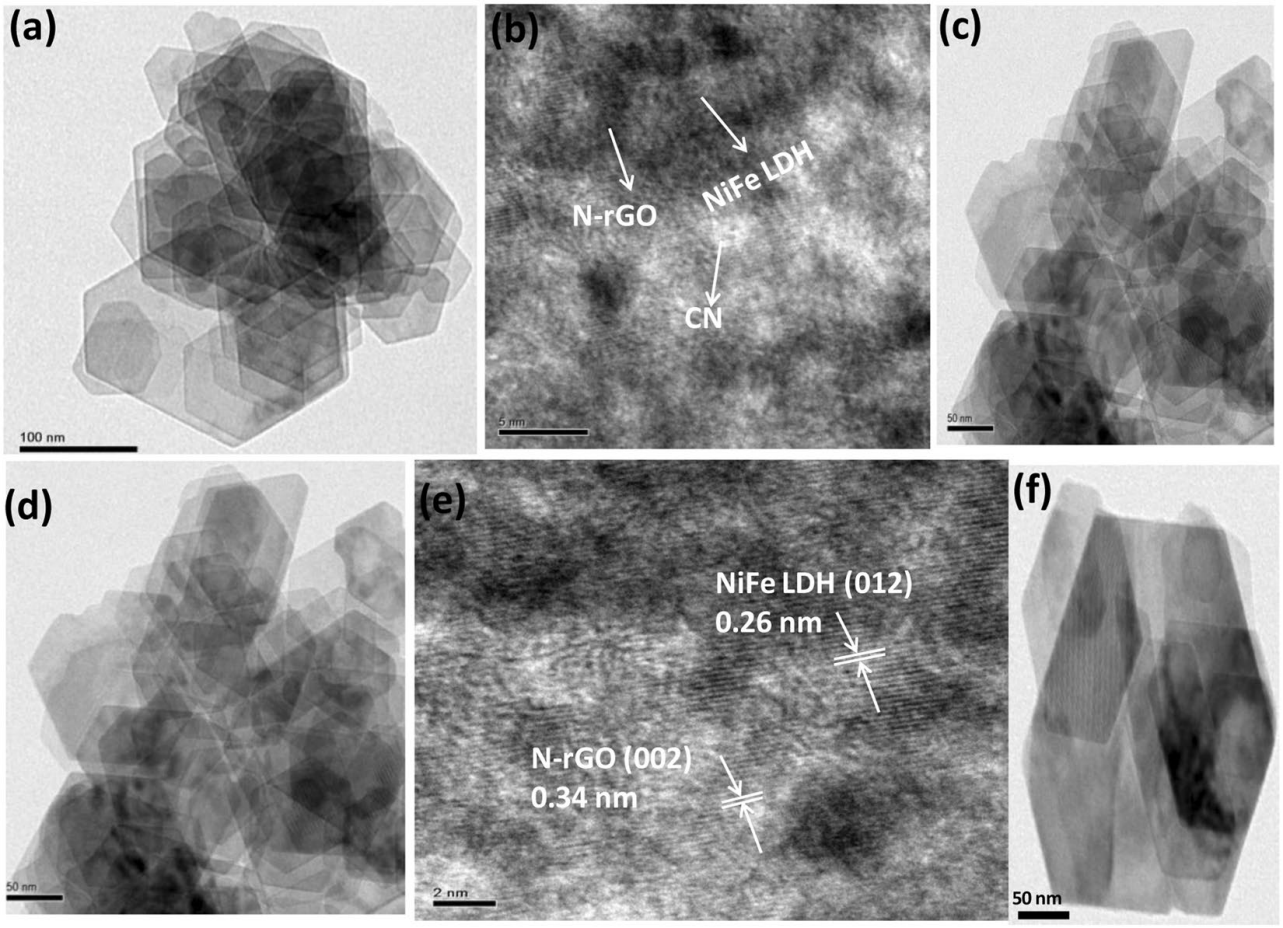
(**a–d**) TEM images of CNNG3LDH, (**e**) HR-TEM images of CNNG3LDH showing lattice fringes of N-rGO and NiFe LDH, and (**f**) TEM image of NiFe LDH.

**Figure 7:**
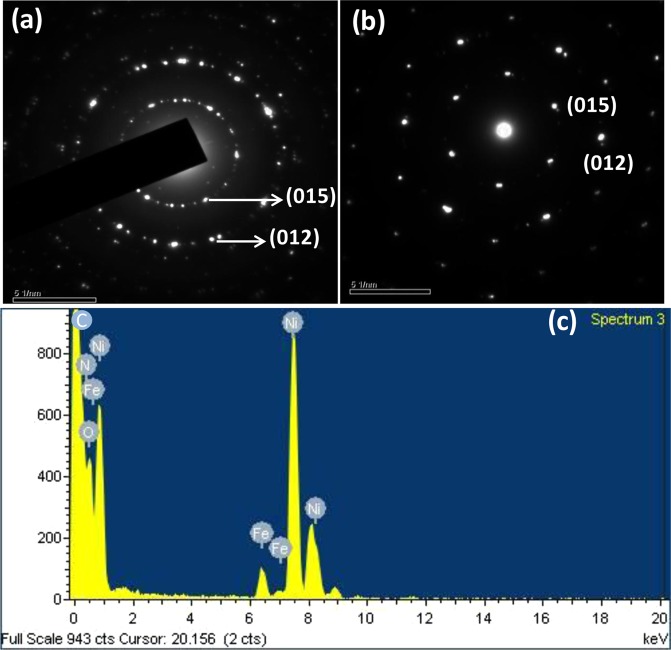
(**a**) SAED pattern, (**b**) FFT pattern and (**c**) EDX spectra of CNNG3LDH.

TEM images of heterostructure CNNG3LDH nanocomposite (Fig. 6a–d) revealed that the heterostructure possesses multiple overlapping of nanosheets in which NiFe LDH and CN were electrostaticaly assembled with the surface of N-rGO framework. The tiny sized pores created on the surface of CN nanosheets by the gas released during the pyrolysis process allows for the efficient scattering of light during photocatalytic reactions (Fig. S2a)^14^. The positively charged exfoliated NiFe LDH nanosheets (Fig. 6f), was grown firmly on the edge of g-C_3_N_4_/N-rGO (Fig. S2c) and adhere to the negatively charged N-rGO sheets (Fig. S2b) through electrostatic interaction and resulted in the formation of heterostructure CNNG3LDH nanocomposite (Fig. 6a–e). The selected area electron diffraction (SAED) and Fourier transformation (FFT) pattern (Fig. 7a,b) of CNNG3LDH consists of hexagonally arranged spots of the exfoliated NiFe LDH nanosheets are still single crystalline and consists of both distorted and undistorted MO_6_ phases without change of the basic layered structure^59^. From the HR-TEM images of CNNG3LDH (Fig. 6e), crystal fringe lattice spacings were measured to be 0.26 and 0.34 nm, which corresponded to the d-spacing value of (012) and (002) planes of NiFe LDH and N-rGO, respectively. By comparing the HR-TEM image of CNNG3LDH with NiFe LDH (Fig. S2d), it could be clearly visualized the distinct lattice fringes corresponding to d(012) and d(002) planes of hexagonal NiFe-LDH and N-rGO, respectively, which indicating that (012) facet of LDH nanosheets interacted with graphene sheet in CNNG3LDH (Fig. 6e). In addition, the incorporation of N-rGO into LDH can prevent LDH nanosheets from aggregation that reflected in terms of clear lattice fringes of NiFe LDH in CNNG3LDH, which improve the specific surface area and provide numerous photochemical active sites of the heterostructure nanocomposite for superior photocatalytic activities.There were no clear crystal lattice fringe of CN found in CNNG3LDH due to its interference with N-rGO^60^. The close interfacial contact among the heterostructure assembly in CNNG3LDH was beneficial for shortening the charge transfer path of excitons pairs, which successfully enhances the photocatalytic activities. The corresponding EDX spectrum of CNNG3LDH (Fig. 7c) clearly demonstrates the spatial distribution of Ni, Fe, C, O and N elements in the heterostructure nanocomposite. A layered area image corresponding to the SEM elemental mapping results of CNNG3LDH (Fig. S3a–f), shows the uniform presence of Ni, Fe, C, O and N with sharp contrast throughout the heterostructure nanocomposite.

### Chemical structure analysis

**Figure 8:**
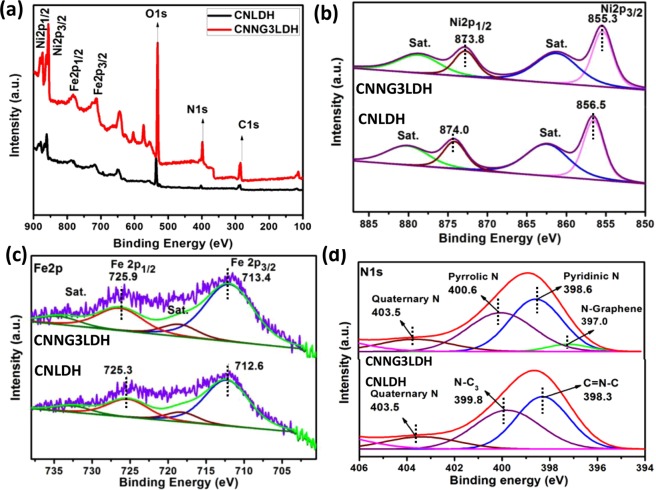
(**a**) Assessment results of the XPS survey spectra of CNLDH with CNNG3LDH and (**b–d**) assessment results of the deconvoluted XPS spectra of CNLDH and CNNG3LDH for Ni2p, Fe2p and N1s.

The X-ray photoelectron spectroscopy (XPS) technique is used to confirm the chemical bonding state of elements and possible chemical interaction between the constituent parts in a heterostructure nanocomposite. Figure 8a displays the XPS spectral survey of the CNLDH and heterostructure CNNG3LDH nanocomposites, which indicating the presence of Ni, Fe, N, O and C with sharp photoelectron peaks at different binding energies. The atomic concentrations for each element of CNLDH determined from the XPS spectra was 54% (C), 7% (N), 31.5% (O), 6% (Ni), and 1.23% (Fe), respectively. Similarly, the atomic concentrations for each element of CNNG3LDH derived from the XPS spectra was 57% (C), 9% (N), 28.0% (O), 5.0% (Ni) and 1.0% (Fe), respectively.The changes in oxidation state of each elements constituted in the as-synthesized materials were further analyzed by deconvoluting the high resolution XPS peak of each connected element using the help of CASA XPS and ORIGIN software.These deconvolution gives enough evidence about the synergistic interactions of component semiconductors in the heterostructure nanocomposite. As in the spectrum of Ni2p, the fitted two spin-orbit doublets of Ni2p3/2 and Ni2p1/2 peaks at 855.3 and 873.8 eV were followed with two prominent shake-up satellites of 861.7 and 879.6, which represents high spin Ni^2+^ state and signify the presence of Ni(OH)_2_ in CNNG3LDH (Fig. 8b)^29,61^. In comparison with the XPS peak centered at 856.5 eV assigned to Ni2p3/2 in CNLDH, negative shifting of 1 eV i.e. 855.3 eV was identified in CNNG3LDH, which suggest the strong chemical interaction of uncoordinated metal centers of exfoliated NiFe LDH nanosheets with highly electron rich N-rGO and g-C_3_N_4_^61^. In sharp contrast, the Fe2p3/2 peak at 713.4 eV and Fe2p1/2 peak at 725.9 eV corresponds to the existence of Fe^+^^3^ oxidation state and verified as Fe(OH)_3_ in CNNG3LDH (Fig. 8c)^61^. Similarly in comparison with the Fe2p3/2 XPS peak of CNLDH i.e. 712.6 eV, positive shifting of ~0.8 eV was identified in CNNG3LDH. The high resolution N1s wide peak of CNNG3LDH could be deconvoluted into four different peaks (Fig. 8d), which could be interpreted as (I) N-graphene-1 (397.0), (II) pyridinic N-2 (398.6 eV), (III) pyrrolic N-3 (399.8 eV), and (IV) chemisorbed nitrogen group such as NH_4_^+^ ions-4 (402.5–405.7 eV), respectively^29,62,63^. It is well known that pyridinic and pyrrolic N centers are highly active site for catalysis that enhances the photocatalytic activities^64^. In comparison with the N 1 s XPS spectrum of N-doped material^63^, and CNLDH, N-graphene-1 (397.0), pyridinic N-2 (398.6 eV) and pyrrolic N-3 (399.8 eV) peak of CNNG3LDH were downshifted, which strongly support the electron transfer from NiFe LDH to CN through N-rGO. Moreover, in comparison with the N1s XPS spectrum of CNLDH, the appearance of N-graphene peak at 397.0 eV in CNNG3LDH shows the doping of N in rGO.

**Figure 9:**
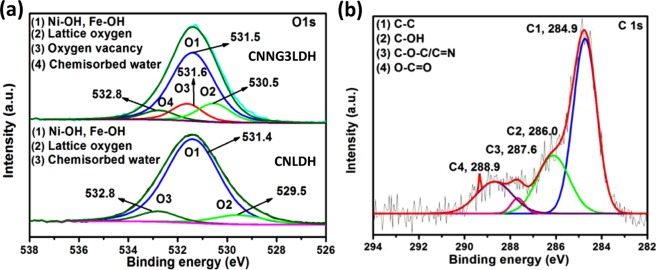
(**a**) Assessment results of the deconvoluted XPS spectra of CNLDH and CNNG3LDH for O1s and (**b**) Deconvoluted XPS spectral curves of CNNG3LDH for C1s.

The high-resolution O1s spectrum of CNNG3LDH (Fig. 9a) reveals four distinct peaks accredited to the surface hydroxyl groups of the metal centre-1 (531.5 eV)^65^, lattice oxygen-2 (530.6 eV)^65^, under-coordinated lattice oxygen of oxygen vacancies-3 (531.6 eV)^66^, and absorbed water-4 (532.8)^67^, respectively. Moreover, compared with the O1s spectrum of CNLDH, the lattice oxygen peak at 530.1 eV was significantly shifted to higher binding energy value ~1 eV in CNNG3LDH. Furthermore, the oxygen vacancies (O3) peak becomes more intact in CNNG3LDH, which proves the existence of oxygen vacancies type defects induced due to the exfoliation of NiFe LDH during the synthesis procedure of CNNG3LDH.This strongly proves the presence of oxygen vacancies with electron transfer from NiFe LDH to CN through N-rGO in heterostructure CNNG3LDH nanocomposite. The deconvolution of C1s XPS spectrum identified as C-C (284.9 eV), C−OH (286 eV), C-O-C/C-N (287.6 eV), and O−C=O (288.9 eV) as shown in Fig. 9b, which yet again confirms the presence of N-rGO^65^. The high resolution C 1 s and N 1 s spectra validate the successful doping of N in rGO and serving as mediator for the effective charge separation between NiFe LDH and CN for excellent photocatalytic activities.

### Electrochemical analysis

**Figure 10:**
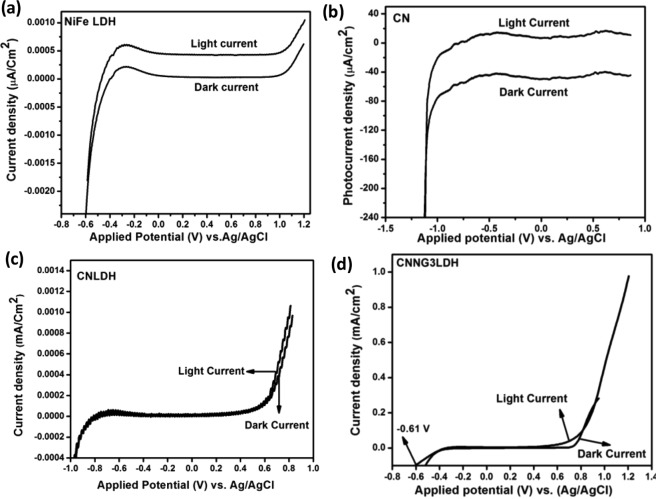
LSV plot of (**a**) NiFe LDH, (**b**) CN, (**c**) CNLDH and (**d**) CNNG3LDH under both dark and light irradiation in aqueous solutions of 0.1 M Na_2_SO_4_ at pH 6.5.

In order to find out the origin of the high photocatalytic activity of the heterostructure CNNG3LDH nanocomposite in comparison to NiFe LDH, CN and CNLDH, LSV, EIS and M-S plots were taken into consideration to investigate the current generation, interfacial charge transfer rate at the electrode/electrolyte interface and majority of carrier density with band edge potential of the material. The electrochemical properties of the as-prepared electrodes (NiFe LDH, CN, CNLDH and CNNG3LDH) were evaluated in a typical three-electrode system in 0.1 M Na_2_SO_4_ aqueous solution as electrolyte. The LSV plots were carried out at scan rate of 0.05 mV s^−1^ under both dark and light illumination (Fig. 10). The LSV curve of pristine NiFe LDH shows photo response behaviour with photocurrent density of 0.0010 µA cm^−2^ at −0.60 V^5,44^. The pristine CN exhibits photocurrent density of 16.40 µA cm^−2^ at potential of −1.13 V^5,29^. Likewise, the CNLDH composite exhibit photocurrent density of 0.0010 mA cm^−2^ at −0.96 V^5,29^. In contrast, CNNG3LDH exhibit highest photocurrent density of 0.97 mA cm^−2^ at lower potential of −0.61 V. These may be due to the phenomenon of strong interfacial contact and co-operative effects of the component in the heterostructure material. Notably, N-rGO provide an sufficient surface area and higher conductivity that well coupled with the other two 2D materials of NiFe LDH and CN, which facilitates fast ion/electron transfer kinetics between the active sites and the contact electrolyte. Moreover, the introduction of N-rGO into NiFe LDH and CN led to considerable cathodic shift in the onset potential of −0.96 V for CNLDH in comparison to −1.13 V for CN and −0.61 V for CNNG3LDH, The cathodic shifting within negative potential indicates availability of both electrons and holes with minimizing charge recombination in the heterostructure. This effect is comparable to that of minimizing the kinetics of overpotential required for water splitting reactions^68–80^. The photocurrent response of pristine NiFe LDH, pristine CN, CNLDH composite and heterostructure CNNG3LDH under dark was very low in comparison to their light current density. However, the current density of CNNG3LDH in the dark is slightly bigger than in the light after 0.8 V (vs. Ag/AgCl), this is due to the creation of nanointerface and charges to move faster in the heterostructure CNNG3LDH electrode at higher potential with direct access of electrolyte, where adsorption of SO_4_^2−^ ions takes place^81^.

**Figure 11:**
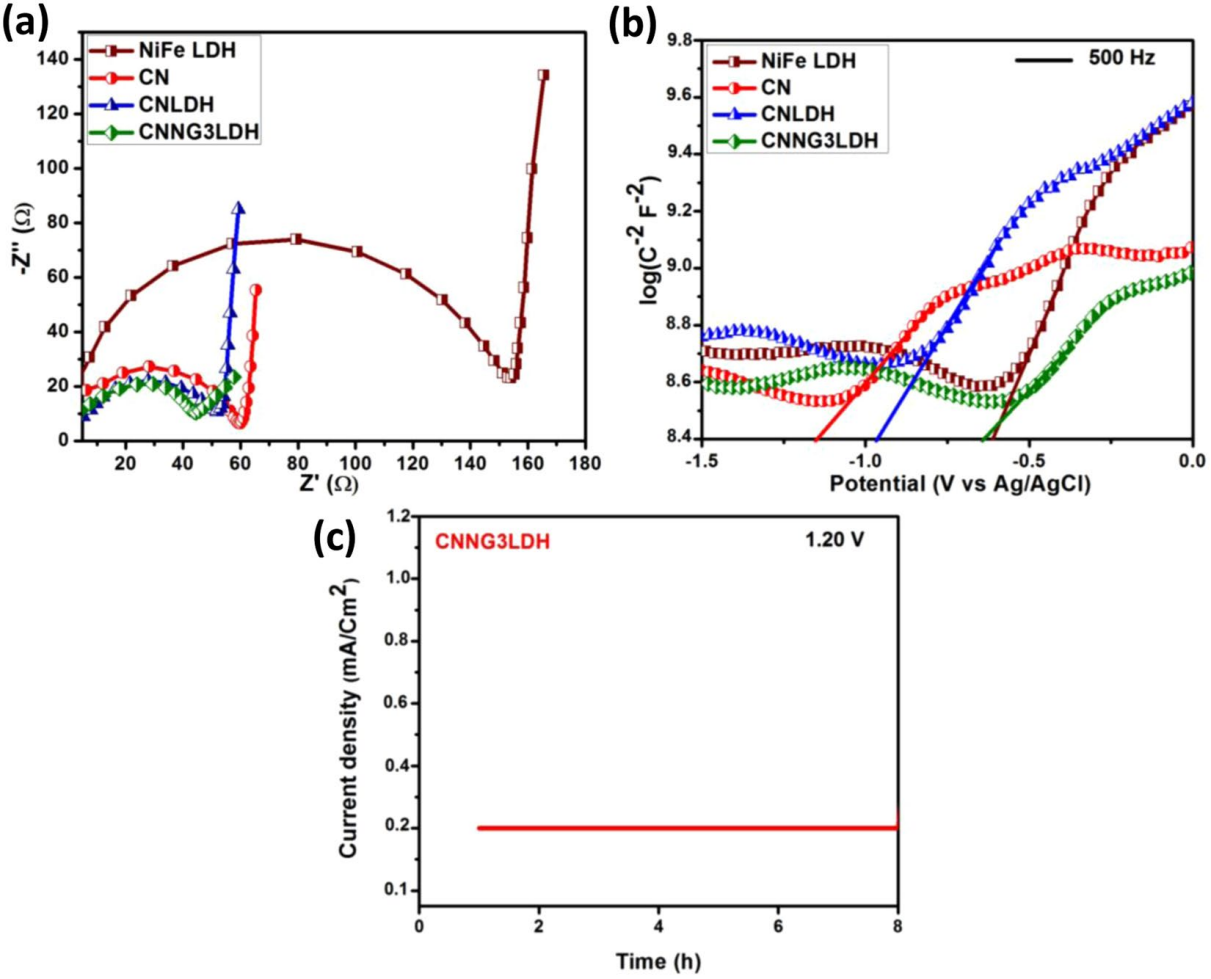
(**a**) Electrochemical impedance spectra (Nyquist plots) of NiFe LDH, CN, CNLDH and CNNG3LDH, (**b**) Mott-Schottky plot of NiFe LDH, CN, CNLDH and CNNG3LDH and (**c**) chronoamperometric study of CNNG3LDH.

To further understand the electrical charge transport kinetics of the heterostructure material, EIS measurements study were carried out in order to reveal the charge transfer resistance (Rct) across the electrode/electrolyte interface. The diameter of the arc radius in the semicircle at high frequency region of electrode material is the measure of charge transfer resistance (Rct) process across the electrode/electrolyte interface^29,30^. The EIS in terms of Nyquist plots of NiFe LDH, CN, CNLDH and CNNG3LDH as prepared electrodes were measured in the frequency range of 10^5^ Hz to 1 Hz at potential of 0.1 V as shown in Fig. 11a. The squeezed diameter of the arc radius of CNNG3LDH indicates that the introduction of N-rGO remarkably reduced the charge transfer resistance and contributed towards shortening of ion diffusion pathway distance and time^65^. The assembly of N-rGO with NiFe LDH and CN at the molecular level facilitates the interfacial charge transfer, where as N-rGO acts as mediator across the interface of NiFe LDH and CN. Moreover, N-rGO has found to be more effective for the well coupling of defective NiFe LDH nanosheets due to larger relative electrochemically active surface area for exposing more active sites. The decreasing order of resistance across charge transfer path of the as prepared electrode could be sequenced as: CNNG3LDH (43.8 Ω) < CNLDH (52.3 Ω) < CN (59.1 Ω) < NiFe LDH (154.2 Ω).

In general, the fundamental principles of M-S measurements were governed by the induced Schottky barrier by the contact of semiconductor electrode surface with electrolyte solution, which was further used to establish the carrier density of a semiconductor material^29,30^. Therefore, the semiconductor properties of NiFe LDH, CN, CNLDH and CNNG3LDH were further investigated by M-S plot analysis as shown in Fig. 11b^82^. The slopes of the M-S plots are very much essential in identifying the properties of n-type semiconductor with positive slope and properties of p-type semiconductor with negative slope inclined towards the X-axis^29,30^. The slopes of the curves in the M-S plots of the as prepared electrodes were found to be positive, which indicate n type semiconductor nature. According to the intercept of linear plot at 1/C^2^ = 0 on E axis gives the flat band potential (*V*_fb_). The *V*_fb_ is approximately equal to the conduction band potential (E_CB_) for n-type semiconductor and valence band potential (E_VB_) for p-type semiconductors^29,30^. The flat-band potentials (E_fb_) of NiFe LDH, CN, CNLDH and CNNG3LDH were calculated to be −0.60, −1.13, −0.96 and −0.61 V *vs*. Ag/AgCl, respectively. Therefore, the E_CB_ potential of NiFe LDH, CN, CNLDH and CNNG3LDH were estimated to be −0.60, −1.13, −0.96 and −0.61 V *vs*. Ag/AgCl, respectively. The cathodic shifting of the E_fb_ of CNNG3LDH as compared to CN and CNLDH strengthened the band bending at the CNNG3LDH/electrolyte interface, which effectively decreases the carrier recombination close to the E_fb_. By comparing the change in E_fb_ of CNLDH before and after the incorporation of N-rGO, it is clear that the N-rGO near the E_fb_ could fasten the water oxidation and reduction kinetics of CNLDH. This reveals that the band bending at the electrode/electrolyte interface induced by the N-rGO and cooperated by the defective NiFe LDH nanosheets leads to larger extent of separation of photogenerated charge carriers. Moreover, the CNNG3LDH shows relatively lesser intense slope than NiFe LDH, CN and CNLDH, which reveals faster charge transfer kinetics^29,30^. The potential (E_CB_) measured relative to Ag/AgCl reference could be converted into normal hydrogen electrode (NHE) potential by using the following Nernst Eq. (4)^29,82,83^.

4 $${{\rm{E}}}_{{\rm{C}}{\rm{B}}}({\rm{NHE}})={{\rm{E}}}_{{\rm{CB}}}({\rm{Ag}}/{\rm{AgCl}})+{{\rm{E}}}^{0}{\rm{Ag}}/{\rm{AgCl}}+0.059\,{\rm{pH}}$$

E^0^Ag/AgCl = 0.197 at 25 °C, and E_CB_ (Ag/AgCl) is the experimentally calculated potential against Ag/AgCl reference electrode and the measured pH value of the 0.1 M Na_2_SO_4_ electrolyte is ~6.5. Therefore, the CB potential of NiFe LDH, CN, CNLDH and CNNG3LDH were estimated to be −0.01, −0.54, −0.37 and −0.02 V *vs*. NHE, respectively. The heterostructure CNNG3LDH nanocomposite intermixes the advantages of enhanced light-harvestation ability, effective separation of carrier charge at the interfacial area, strong coupling effect with shortening of transport time and improved surface reaction kinetics for the multifaceted photocatalytic performances.

Stability of electrode is another vital factor to determine the quality of a catalyst. The chronoamperometric study of CNNG3LDH (Fig. 11c) revealed that around 95% of the original current density was maintained after 8 h of irradiation at 1.20 V showing its excellent stability for photocatalytic reactions.

### Photocatalytic activity assessment

#### RhB dye and Phenol degradation study

**Figure 12:**
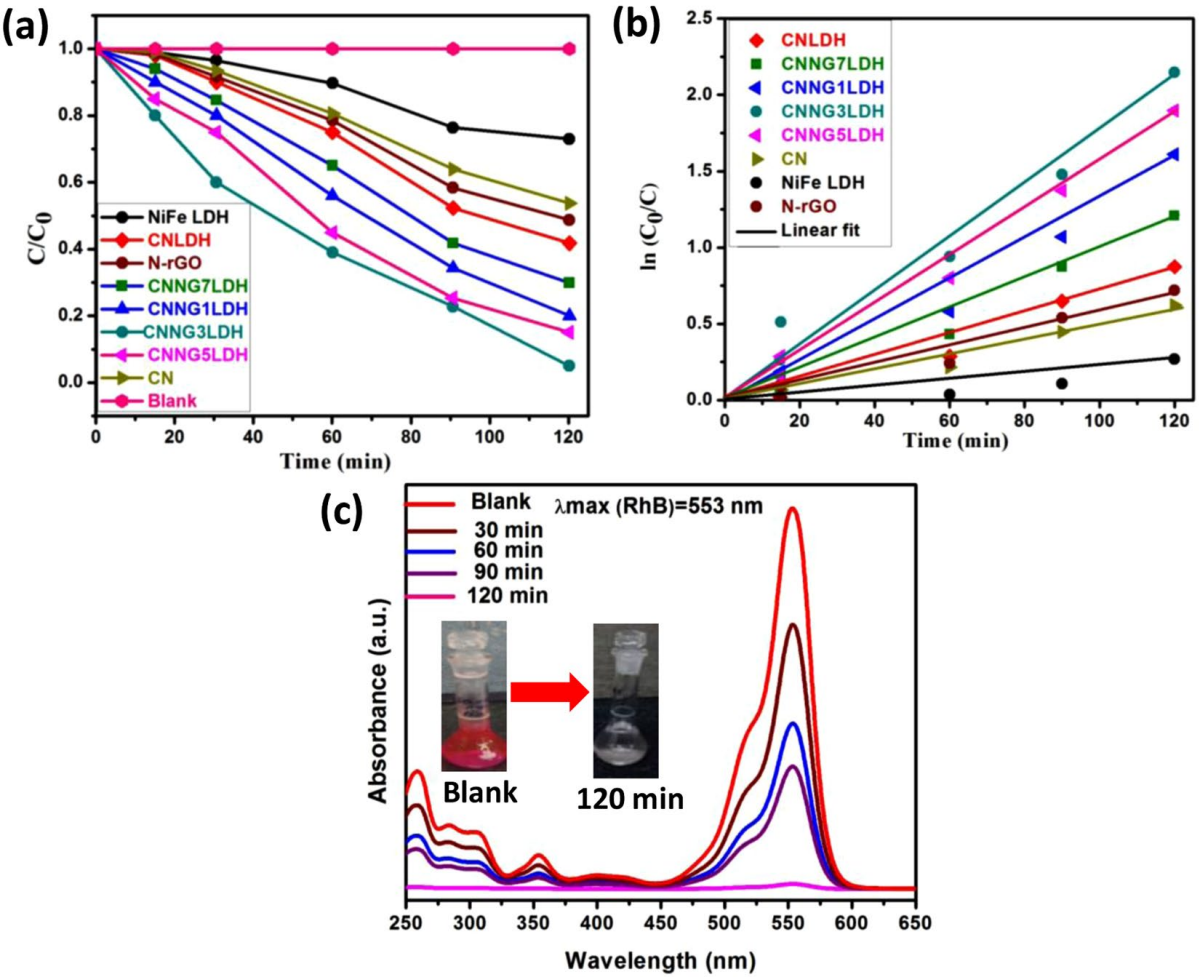
(**a**) C/C_0_ *vs*. time plot of RhB degradation and (**b**) Kinetics of RhB degradation with NiFe LDH, CN, N-rGO, CNLDH and CNNGxLDH, (**c**) UV–Vis absorbance spectral changes of RhB as function of time over CNNG3LDH.

For RhB dye degradation, the photocatalytic reactions were carried out by suspending 0.02 g of catalyst in 20 ppm of 20 mL of RhB dye solution under natural sun light exposure. Almost no changes in the concentration of RhB dye was noticed without the presence of catalyst or sun light irradiation for about 2 h, which indicates that self-degradation of RhB dye was almost negligible and the photodegradation was caused by the presence of both photocatalyst and light. The adsorption of pollutants over the catalyst surface is the primary step for photocatalytic degradation. Prior to degradation study, dark adsorption of RhB dye solution was measured for 30 min to reach an adsorption–desorption equilibrium (Fig. S4). The adsorption rate was found to be increased from 38.5% (CNNG1LDH) to 45.5% (CNNG3LDH) and then decreases 40.0% (CNNG5LDH) to 32.2% (CNNG7LDH). At the same time, the adsorption rate of NiFe LDH, CN and CNLDH were measured to be 14, 20 and 25%, respectively. The amount of dye adsorbed at equilibration time was calculated by using Eqs (7, 8) and the values were found to be 7.7 mg/g (CNNG1LDH), 9.1 mg/g (CNNG3LDH), 8 mg/g (CNNG5LDH), 6.44 mg/g (CNNG7LDH), 5 mg/g (CNLDH), 4.4 mg/g (N-rGO), 4 mg/g (CN) and 2.8 mg/g (NiFe LDH), respectively. After sun light exposure of 120 min, 30% and 47%of RhB dye were degraded over NiFe LDH and CN, respectively. For instance, CNLDH shows enhanced photocatalytic degradations of RhB as compared to NiFe LDH and CN. The degradation rates of CNLDH reached to 60% due to the layered to layered coupling and synergistic effect of both LDH and CN, which effectively improve the anti-recombination of charge carriers. The RhB degradation rate of N-rGO was tested to be 52%. In heterostructure CNNGxLDH nanocomposite, the incorporation of N-rGO into NiFe LDH nanosheets and CN attains Z-scheme mechanistic path, which restores the electrons and holes on their higher reduction and oxidation potentials of CN and LDH, respectively while extra excitons were recombined over N-rGO surface^64^. These results an increase in degradation rate of 81% (CNNG1LDH) to 97% (CNNG3LDH) and then decreases to 85% (CNNG5LDH) to 71% (CNNG7LDH) due to the black body radiation effect of N-rGO in the heterostructure CNNGxLDH. The excess loading of black colored N-rGO in CNNGxLDH, x = 5 and 7 wt% might shield the active sites on the surface of catalyst and decrease the light intensity penetration into the depth of the reaction solution^64^. Therefore, control over the content of N-rGO is very much crucial during the photocatalytic activity evaluation of CNNGxLDH. However, CNNG3LDH demonstrate the highest photocatalytic degradation activity under sun light illumination. The photo-degradation rate was determined by plotting C/C_0_ *vs*. time (Fig. 12a) using the following Eq. (5).

5 $${\rm{Photo}} \mbox{-} {\rm{degradation}}\,{\rm{rate}}=({{\rm{C}}}_{0}-{\rm{C}}/{{\rm{C}}}_{0})\times 100$$

where C_0_ is the initial concentration at time t = 0 min C is the concentration at time’t’ min. For better quantitative understanding of the reaction kinetics of the synthesized heterostructure, kinetic analysis of the degradation of RhB dye was carried out under sun light irradiation using the Langmuir–Hinshelwood model Eq. (6) as follows:

6 $$\mathrm{ln}\,({{\rm{C}}}_{0}/{\rm{C}})={{\rm{k}}}_{{\rm{app}}}{\rm{t}}$$

where k_app_ is the apparent rate constant. The kinetics of the rate constant is calculated by the linear plot between ln (C_0_/C) *vs*. irradiation time as shown in Fig. 12b. The slope of the regression curve of ln (C_0_/C) *vs*. irradiation time gives the value of k or k_app_. The regression coefficient (R^2^) values for the heterostructure were listed in Table S2. The k_app_ values for the degradation of RhB dye follow pseudo-first order kinetics of the Langmuir−Hinshelwood model for the NiFe LDH, CN, CNLDH and heterostructure CNNGxLDH (Table S2). The improved k_app_ value in CNNG3LDH verifies its dynamic nature as a heterostructure photocatalyst towards RhB dye degradation. Figure 12c shows the temporal changes of absorption spectra of RhB dye catalyzed by heterostructure CNNG3LDH under the exposure of sun light. The spectra shows a decrease in absorption intensity of RhB as the exposure time increases from 0 to 120 min without any shifting in absorption band. Here, we could observe certain percentage of mineralization of degradation of dye molecules by the heterostructure CNNG3LDH devoid of any harmful side products.

Generally, the reactive species involved in the degradation of organic pollutant were preceded by the participation of holes (h^+^), hydroxyl radicals (^•^OH), superoxide radicals (^•^O_2_^−^) and electrons (e^−^), respectively. Specifically, p-benzoquinone (p-BQ), DMSO, IPA and EDTA acts as scavenging reagents for superoxide radicals, electrons, hydroxyl radicals and holes for the drop down of RhB during degradation process. For carrying out the quencher experiments, 5 mM of each scavenger were added to 20 ppm solution of RhB dye. Figure S5a shows the active species that were involved in RhB dye degradation process. Also, Fig. S5a shows a drop in photodegradation activities of 23%, 36%, 52% and 88% for p-BQ, EDTA, IPA and DMSO, respectively. Therefore, the key reactive species mainly responsible for the degradation of pollutants includes ^•^O_2_^−^, h^+^ and ^•^OH, respectively. When DMSO was used as trapping agent for electrons, the degradation rate was near about to CNNG3LDH (without scavenger), which denotes that electrons played minor role in degradation of RhB.

The identification of ^•^O_2_^−^ radicals were studied by ESR. By that technique, NiFe LDH, CN and CNNG3LDH were subjected to thermal treatment in vacuum at 473 K, followed by exposure to an oxygen environment at room temperature prior to ESR measurements. As observed, the production of ^•^O_2_^−^ radicals in the reaction system could be detected by the ESR technique^84^. Fig. S5b shows that the characteristic peaks of DMPO–^•^O_2_^−^ adducts for NiFe LDH, CN and CNNG3LDH samples after irradiation. Furthermore, higher peaks intensity of CNNG3LDH than CN indicates that the amount of ^•^O_2_^−^ radicals produced on the surface of CNNG3LDH was more than that of CN. However, the intensity of characteristic peak of DMPO–^•^O_2_^−^ for NiFe LDH was very lower than other samples. It demonstrate that no ^•^O_2_^−^ radicals were generated on the CB edge of NiFe LDH. Finally, ^•^O_2_^−^ and ^•^OH radicals were produced on CN, while only h^+^ radicals were generated on NiFe LDH. When both NiFe LDH and CN were coupled with N-rGO, h^+^, ^•^O_2_^−^ and ^•^OH radicals were produced on CNNG3LDH surface for superior photocatalytic activities.

Furthermore the presence of ^•^OH radical was recognize by typical experiment using terephthalic acid photoluminescence probing technique (TA-PL). These terephthalic acid molecules reacts with ^•^OH radical to produce fluorescence active product i.e. 2-hydroxyterephthalic acid with an emission peak at 426 nm at an excitation energy of 315 nm. The probe experiment was carried out by adding each of the as synthesized NiFe LDH, CN, CNLDH and CNNGxLDH samples (0.02 g) to an aqueous solution containing 20 mL of 4 × 10^–3^ M NaOH solution of terephthalic acid. The suspension was illuminated for 2 h under sun light, and the fluorescence spectrum of the transparent solution was taken for spectral analysis. The intensity of fluorescence spectrum of 2-hydroxyterephthalic acid is proportional to the amount of ^•^OH radical produced under visible light irradiation. The TA-PL spectra (Fig. S5c) shows the highest fluorescence intensity of CNNG3LDH, which indicate the larger quantity of generation of ^•^OH radicals.

**Figure 13:**
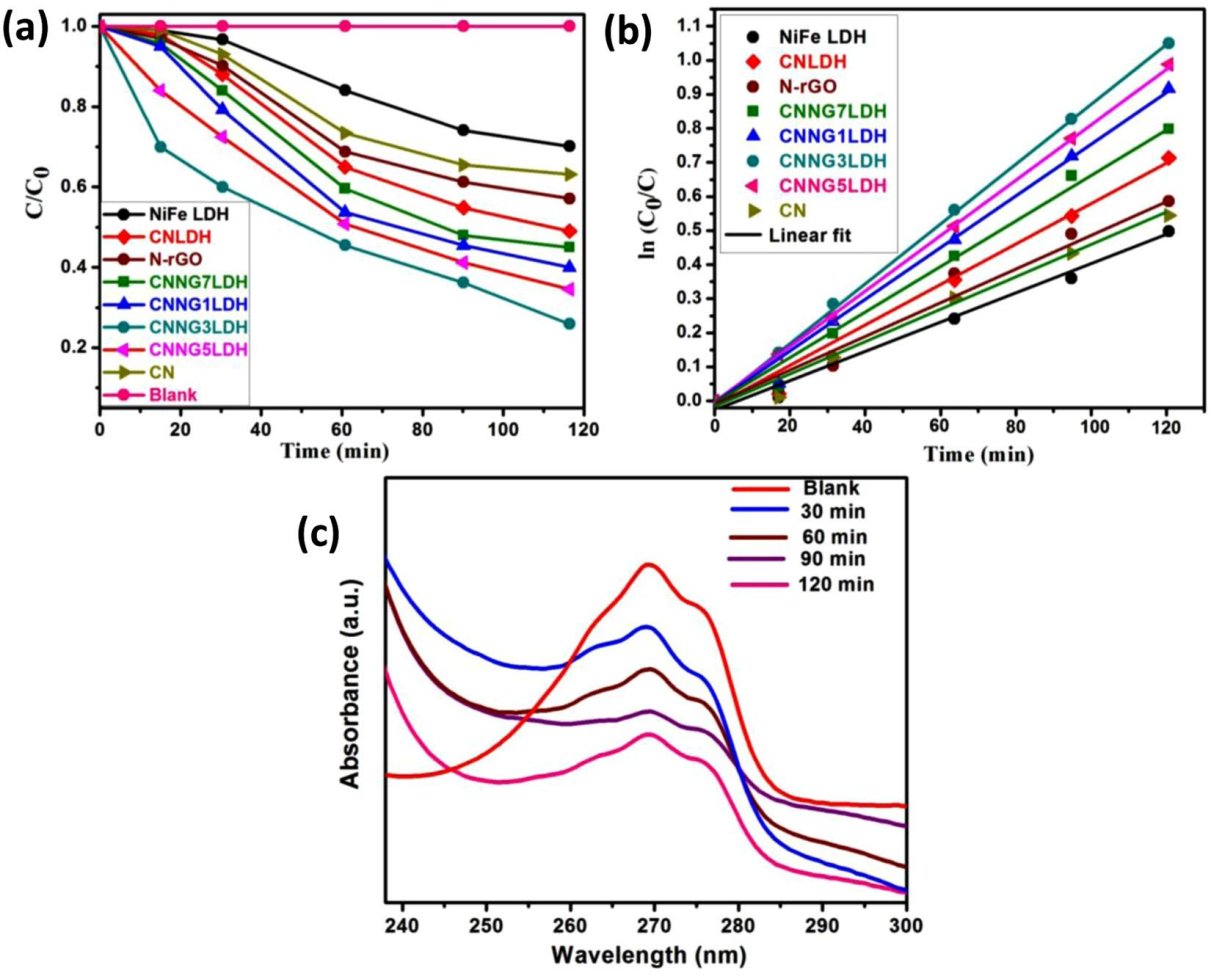
(**a**) C/C_0_ *vs*. time plot of phenol degradation with NiFe LDH, CN, N-rGO, CNLDH and CNNGxLDH, (**b**) Kinetics of phenol degradation over NiFe LDH, CN, N-rGO, CNLDH and CNNGxLDH and (**c**) UV–Vis absorbance spectral changes of phenol as function of time over CNNG3LDH.

The photocatalytic degradation reaction of phenol was carried out by using 20 mL of 20 ppm phenol solution and 0.02 g of catalyst reacted for 120 min in the presence of natural sun light. As shown in Fig. 13a, 27% and 37% of phenol were degraded for NiFe LDH and CN, respectively. CNLDH showed 51% of degradation. The phenol degradation rate of N-rGO was tested to be 42%. Alternatively, the degradation trend of heterostructure CNNGxLDH follows the order as: CNNG7LDH (56%) < CNNG1LDH (60%) < CNNG5LDH (66%) < CNNG3LDH (75%). The heterostructure CNNG3LDH shows highest phenol degradation rate with 75% in 2 h, which indicate the maximum role of N-rGO in enhancing the activity of CNNG3LDH. The kinetics of degradation plots of phenol were shown in Fig. 13b. There were no degradation noticed either in the absence of photocatalyst or light. The degradation rate of phenol follows pseudo first order kinetics with rate constants and regression coefficient listed in Table S3. For the heterostructure CNNGxLDH, presence of N-rGO acts as mediator between NiFe LDH and CN following Z scheme mechanism path for charge separation, where both NiFe LDH and CN with their holes and electrons at respective higher oxidation and reduction potential, respectively. Therefore, the photoinduced electrons generated over the CB of CN in CNNG3LDH capture the dissolved oxygen to form ^•^O_2_^−^ and further to ^•^OH, which possess very strong oxidizing ability to oxidize phenol to p-benzoquinone and subsequent mineralization. The photoinduced holes generated over the VB of NiFe LDH produced ^•^OH, which participate in mineralization of phenol. The spectral changes of concentration of phenol as a function of time were shown in Fig. 13c. The intensity of absorption spectra of phenol at 269.5 nm grow fainter and the simultaneous appearance of absorption peak of some intermediates aromatic compounds such as catechol (278–280 nm) and p-benzoquinone (249 nm) were then mineralizes to CO_2_ and H_2_O. These results indicate that the heterostructure CNNG3LDH could selectively oxidizes phenol to catechol and p-benzoquinone under natural sun light irradiation^85^. Morphology of interconnected nanosheets of heterostructure CNNG3LDH have profound effect on phenol degradation, This is because of high aspect ratio and quantum confinement effect, which increases the morphological stability and enhances the activities.

It is generally known that some intermediate products of degradation are highly carcinogenic and are threat to the environment. Therefore, total organic carbon (TOC) measurements were carried out to establish the mineralization of RhB and phenol. A complete disappearance of organic carbon percentage would corroborate the complete mineralization of organic pollutants. Figure S6 shows the normalized TOC plots *vs*. irradiation time for RhB and phenol. After 2 h of irradiation, TOC removal of RhB and phenol over CNNG3LDH were about 95% and 72%. However, the main absorption peaks of RhB after 120 min of irradiation approximately disappear due to chromophoric reduction by reactive species and thereby facilitate the mineralization to 95%.

### Photocatalytic H_2_ and O_2_ production activities

**Figure 14:**
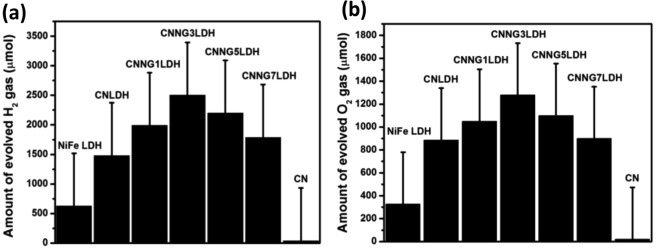
Temporal changes of the (**a**) amount of H_2_ evolved (µmol/g) and (**b**) amount of oxygen evolved (µmol/g) over NiFe LDH, CN, CNLDH and CNNGxLDH.

The photocatalytic H_2_ production of NiFe LDH, CN, CNLDH and heterostructure CNNGxLDH were carried out in 30 mL aqueous CH_3_OH solution under visible-light irradiation (ʎ ≥ 400 nm) as shown in Fig. 14a. There were no H_2_ production was detected without light or catalyst, which suggest that photocatalytic H_2_ production reactions were driven together with light as well as catalyst. The NiFe LDH exhibit H_2_ production rate of 633 µmolg^−1^2h^−1^ and CN showed 46 µmolg^−1^2h^−1^^5^. However CNLDH composite showed 1488 µmolg^−1^2h^−1^^5^. Interestingly, the CNNGxLDH operated via the Z-scheme carrier transfer mechanism between NiFe LDH and CN, mediated by N-rGO exhibit superior H_2_ production activity than NiFe LDH, CN and CNLDH. When the N-rGO wt% varies from 1 to 3 in CNNGxLDH, the rate of H_2_ production increases from 1996 µmolg^−1^2h^−1^ (CNNG1LDH) to 2508 µmolg^−1^2h^−1^(CNNG3LDH) and then decreases to 2214 µmolg^−1^2h^−1^ (CNNG5LDH) and 1794 µmolg^−1^2h^−1^ (CNNG7LDH). The high transparent and conductive nature of N-rGO sheets leads to high mobility of charge carriers and increases the active sites for H_2_ evolution reaction in heterostructure CNNGxLDH. Therefore, CNNG3LDH produces majority of H_2_ at about 2508 µmolg^−1^2h^−1^ with maximum separation of excitons pair. However, over loading of N-rGO resulted in the decreasing H_2_ production rates, which could be attributed to the shielding effect of N-rGO. In case of the photocatalytic O_2_ production reaction of NiFe LDH, CN, CNLDH and heterostructure CNNGxLDH (Fig. 14b), 30 mL aqueous AgNO_3_ solution was used as electron scavenger. There were no O_2_ gas evolution detected in the absence of either catalyst or light irradiation, which indicate that O_2_ production dependence by photocatalysis and sacrificial agents. NiFe LDH exhibit O_2_ production rate of 327 µmolg^−1^2h^−1^^5^. CN and CNLDH exhibit O_2_ production rate of 20 µmolg^−1^2h^−1^ and 886 µmolg^−1^2h^−1^, respectively^5^. However in heterostructure CNNGxLDH, photocatalytic O_2_ production follows the trend as: CNNG7LDH (900 µmolg^−1^2h^−1^) < CNNG1LDH (1050 µmolg^−1^2h^−1^) < CNNG5LDH (1100 µmolg^−1^2h^−1^) < CNNG3LDH (1280 µmolg^−1^2h^−1^). The superior O_2_ production activities of heterostructure CNNG3LDH with 1280 µmolg^−1^2h^−1^ was due to the presence of N-rGO based mediator for separating excitons pairs between NiFe LDH and CN. In this charge separation process, O_2_ production was detected at the VB edge of highly oxidative NiFe LDH. Furthermore, an excessive loading of N-rGO results in decrease in photocatalytic activity due to the blackbody scattering effect of N-rGO, which could obstruct the absorption of light to the catalyst suspension as found in CNNG7LDH^29,30,64^.

**Figure 15:**
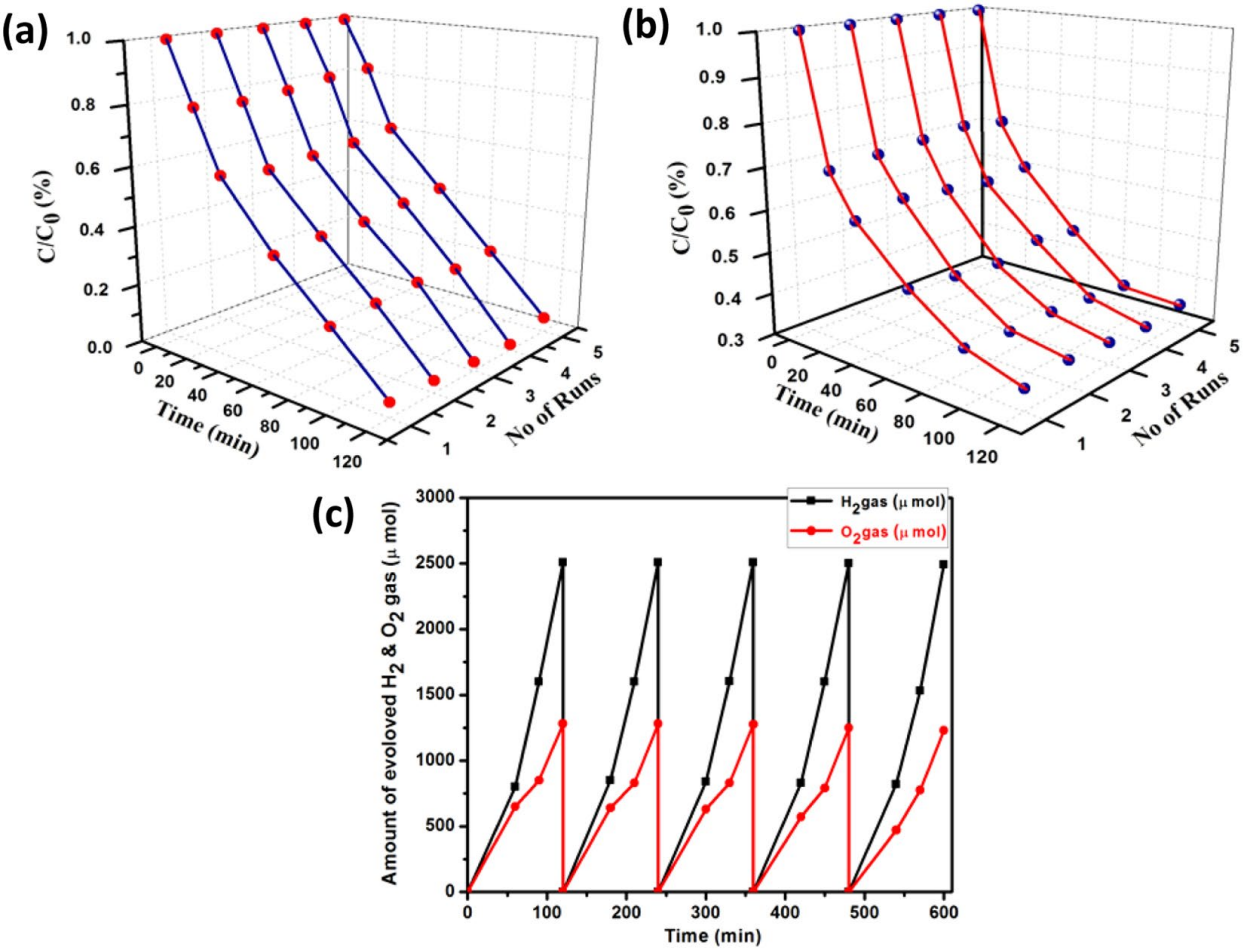
Recyclability study of (**a**) RhB, (**b**) Phenol and (**c**) volume of H_2_ and O_2_ evolved in five consecutive cycles of run in every 120 mins of time interval over CNNG3LDH.

It is very much crucial to test the recyclability or service lifetime of a photocatalyst for real time application as its efficiency may change after the interaction with a particular pollutant or sacrificial agents in an aqueous medium under sun light or visible light irradiation. To confirm the photostability and reusability of the catalyst for practical applications value, heterostructure CNNG3LDH were used for five different cycles for RhB and phenol with H_2_ and O_2_ production activities. Figure 15a,b, reveals that heterostructure CNNG3LDH could maintain excellent photocatalytic activity even after repeated use of five cycles, which confirms its high stability and reusability for the RhB dye and phenol polluted waste water treatment. Moreover, the catalyst recyclability test for H_2_ and O_2_ production performance remain constant with the irradiation time without any deactivation in repetitive 5^th^ cycle as shown in Fig. 15c. After the recyclability test, XRD and XPS analyses were carried out to further confirm the stability of the heterostructure CNNG3LDH nanocomposite. There were no obvious phase changes on the XRD pattern after H_2_ evolution and RhB degradation reactions (Fig. S7). XPS spectra taken after H_2_ evolution and RhB degradation reactions, indicated that Ni2p, Fe2p, N1s, O1s and C1s still show their characteristic peaks in CNNG3LDH (Fig. S8a–e). All these experiments indicated that CNNG3LDH catalyst possesses an excellent stability for practical applications. A state of comparison of photocatalytic activities of CNNG3LDH with other reported materials were well mentioned in Table S4-S7.

### Insight of Z-Scheme charge separation path in heterostructure CNNG3LDH nanocomposite

**Figure 16:**
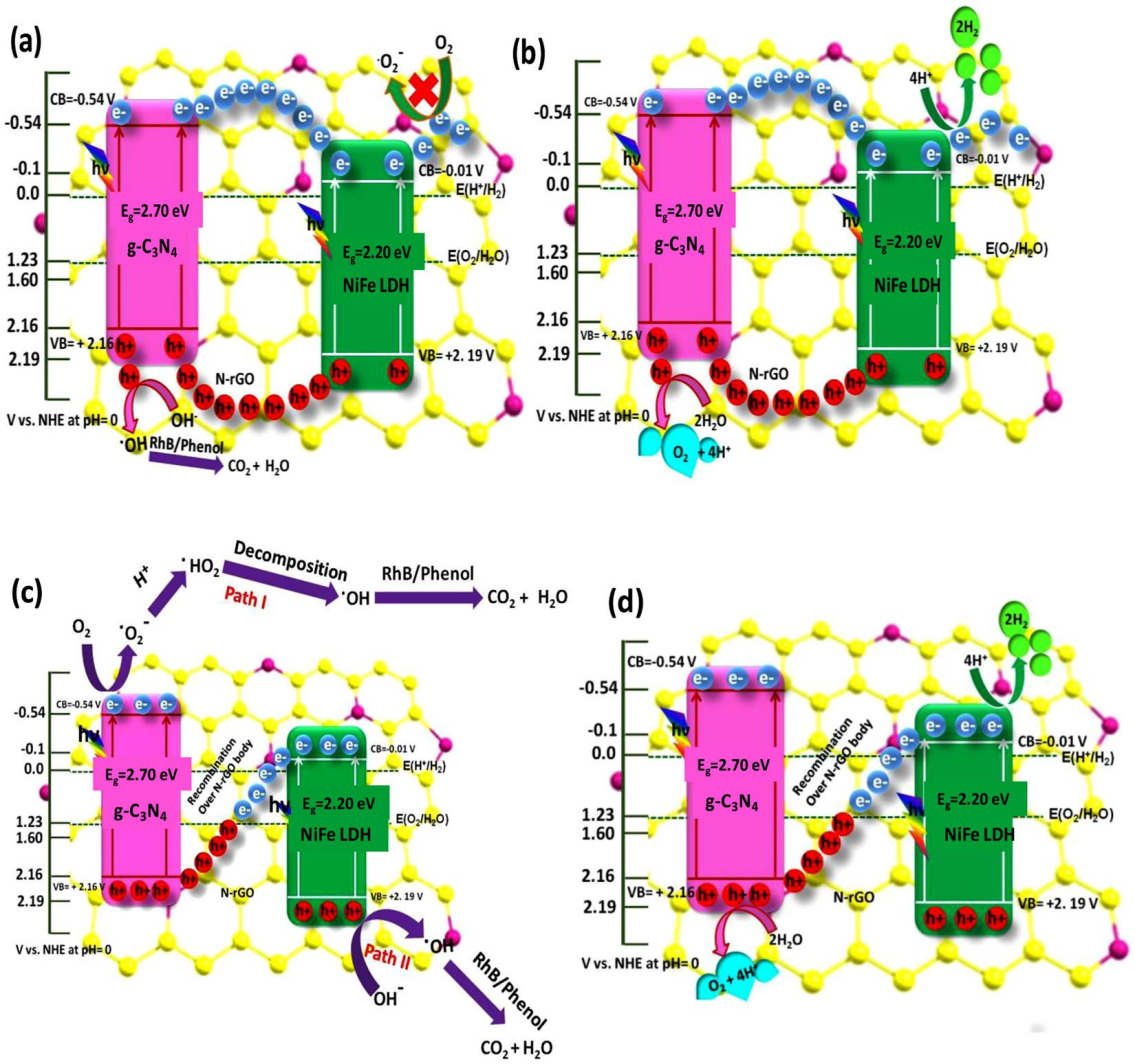
Proposed schematic representation of (**a**,**b**) conventional double charge transfer mechanism and (**c**,**d**) N-rGO mediator based Z-scheme mechanism for mineralization of dyes with H_2_ and O_2_ evolution over CNNG3LDH.

To further analyze the fundamental aspect based on results and discussion section, possible Z-scheme charge transfer mechanistic path for the heterostructure CNNG3LDH nanocomposite was proposed and compared with direct charge transfer route between NiFe LDH and CN (Fig. 16) in order to prove the role of N-rGO as solid mediator in shortening the charge transport time and promoting carrier separation efficiency. The whole photocatalytic process is initiated by the absorption of photon energy equal to or higher than the band gap energy of the constituent semiconductor, which result in the generation of charge pairs under visible-light irradiation. Both the CB and VB edge potential of NiFe LDH (E_CB_ = −0.01 V, E_VB_ = +2.19 V *vs*. NHE) and CN (E_CB_ = -0.54 V, E_VB_ = +2.16 V *vs*. NHE) were excited to generate photoinduced charge carriers. In direct electron–hole transfer between NiFe LDH and CN, the photoinduced electrons on the CB of CN would transfer to the CB of NiFe LDH, whereas the photoinduced holes in the VB of NiFe LDH would transfer towards the VB of CN (Fig. 16a-b)^5^. In this way of charge pair separation, electrons on the CB of NiFe LDH further migrated towards N-rGO surface and couldn’t be able to produce ^•^O_2_^−^ and ^•^HO_2_ radicals owing to more positive CB edge potential of NiFe LDH (-0.01 V *vs*. NHE) than the standard redox potential of E^Θ^ (O_2_/^•^O_2_^−^, −0.33 eV *vs*. NHE) and E^Θ^ (O_2_/^•^HO_2_, −0.05 eV *vs*. NHE), respectively^62,64,84^. In addition, the VB potential of CN (+2.16 V *vs*. NHE) is more negative than the VB potential of NiFe LDH (+2.19 V *vs*. NHE), which suggest that hole in the VB of CN to oxidize OH^−^ to ^•^OH is in a lower potential than NiFe LDH. Nevertheless, if the carriers charge transfer process is in accordance with the direct charge transfer mode (Fig. 16a), it would not be favorable for the construction of a Z-scheme system as well as generation of the main reactive species (^•^O_2_^−^ and ^•^OH) for superior photocatalytic activity. Similarly, the photocatalytic H_2_ and O_2_ production activity as per direct charge transfer route between NiFe LDH and CN were preceded at lower reduction and oxidation reaction potential edge (Fig. 16b). Therefore, the photoexcited charge carrier separation process is thermodynamically more favorable with the introduction of N-rGO in CNLDH resulted as heterostructure CNNG3LDH as per proposed Z–scheme mechanistic path rather than direct contact between NiFe LDH and CN (Fig. 16c,d). The band alignment level at the CB and VB edge potential of CNNG3LDH were thermodynamically more favorable for photocatalytic organic pollutant degradation as well as water splitting reactions for the production of H_2_ and O_2_. The NiFe LDH nanosheets electrostaticaly bonded with the N-rGO surface in CNNG3LDH could form good Schottky junctions by contacting defect site of NiFe LDH with N-rGO and the photogenerated electrons at the CB edge of NiFe LDH could quickly transferred to the N-rGO surface under visible light irradiation^64^. In the meanwhile, the strong electrostatic bonding interaction of CN with NiFe LDH could form a good contact with the N-rGO surface, where the photogenerated holes on the VB of CN could directly recombine with the photogenerated electrons on the CB of NiFe LDH over the N-rGO surface. Moreover, these charge transfer routes represent kinetically more favorable strategy in comparisons with the direct electron-hole transport route between NiFe LDH and CN in CNLDH. In heterostructure CNNG3LDH, photoinduced electrons with higher reduction abilities and photoinduced holes with strong oxidation abilities were accumulated on the CB and VB positions of CN and NiFe LDH, respectively. Besides, due to the unique features of CNNG3LDH, N-rGO could function as solid electron mediator to lengthen the lifetime of charge carriers, where photogenerated electrons from the CB of NiFe LDH could be efficiently transferred to N-rGO surface^23–26,64^. Moreover, the presence of defects site as oxygen vacancies in NiFe LDH could trap the photogenerated charge carriers promoting further charge separation in CNNG3LDH. In CNNG3LDH, the accumulated electrons on the CB edge potential of CN (-0.54 V *vs*. NHE) is more negative than the standard redox potential of E^Ѳ^(O_2_/^•^O_2_^−^ = −0.33 eV *vs*. NHE) and E^Ѳ^ (O_2_/^•^HO_2_ = −0.05 eV *vs*. NHE). Therefore, the electrons subsequently escape to the surface of CN and have sufficient potential to pick up dissolved oxygen to form superoxide radicals E^Ѳ^ (O_2_ /^•^O_2_^−^ = −0.33 eV *vs*. NHE). In path I of Fig. 16c, the ^•^O_2_^−^ radicals would directly oxidize the pollutants into nontoxic products or the reaction of ^•^O_2_^−^ radicals with H^+^ to produce hydroperoxy radical E^Ѳ^ (O_2_/^•^HO_2_ = −0.05 eV *vs*. NHE), which subsequently decomposed to ^•^OH active species and further mineralize the degraded products to CO_2_ and H_2_O^62,63,84^. In path II, the VB of NiFe LDH (+2.19 eV *vs*. NHE) is in a more positive potential than the redox potential of E^Ѳ^ (^•^OH /OH^−^ = +1.99 eV *vs*. NHE) to produce ^•^OH radicals. Therefore, the holes on the VB of NiFe LDH could react with H_2_O to produce the ^•^OH radicals, which subsequently mineralize degraded products to CO_2_ and H_2_O (Fig. 16c). Similarly, for photocatalytic H_2_ and O_2_ production (Fig. 16d), the electron enriched VB of CN (−0.54 V *vs*. NHE) could directly reduce H^+^/H_2_O to produce H_2_. Likewise, the hole enriched VB of NiFe LDH (+2.19 V *vs*. NHE) oxidized H_2_O to produce O_2_ gas.

### Evidence in support of Z-scheme mechanism in heterostructure CNNG3LDH

To confirm the Z-scheme mechanism of carrier charge separation in CNNG3LDH, ESR experiments were carried out to detect the superoxide radicals (DMPO–^•^O_2_^−^) and the results were shown in Fig. S5b. There were no signals of ^•^O_2_^−^ detected in the spectra of NiFe LDH. However, the signals of DMPO–^•^O_2_^−^ adducts were detected in the ESR spectra of CN and CNNG3LDH. These results demonstrate that the ^•^O_2_^−^ have been produced at the CB of CN in CNNG3LDH at higher reduction potential during photocatalysis. The accumulated electrons on CB edge potential of CN (−0.54 V *vs*. NHE) is more negative than the standard redox potential of E^Ѳ^ (O_2_/^•^O_2_^−^ = −0.33 eV *vs*. NHE) and E^Ѳ^ (O_2_/^•^HO_2_ = −0.05 eV *vs*. NHE). Therefore, the photogenerated electrons of CN have sufficient potential to pick up dissolved oxygen to form superoxide radicals E^Ѳ^ (O_2_ /^•^O_2_^−^ = −0.33 eV *vs*. NHE). Alternatively, there were almost no ^•^O_2_^−^ radicals produced at the CB edge potential of NiFe LDH owing to higher positive CB edge potential (−0.01 V *vs*. NHE) than E^Ѳ^ (O_2_/^•^O_2_^−^ = −0.33 eV *vs*. NHE). These results reveal that the photoexcited electrons of CN were not transferred from the CB of CN to the CB of NiFe LDH. According to the ESR results, the transfer of photoexcited charge carriers in CNNG3LDH follows Z-scheme mechanistic pathway in accordance with Fig. 16c,d.

Furthermore, ^•^OH radical trapping test is one of the most common methods to confirm the heterostructure formation following Z-scheme mechanistic path of carrier charge separation at the interfacial region. The terephthalic acid photoluminescence probing technique (TA-PL) was applied to investigate the ^•^OH radicals since TA could react with ^•^OH radicals to from a highly fluorescent 2-hydroxyterephthalic acid (TAOH) with emission band at around 425 nm. The peak intensity increases with the increase in irradiation time, which indicate that higher rate of formation of ^•^OH radicals by the said photocatalytic material. As shown in Fig. S5c, it could be found that highest fluorescence emission intensity was detectable for CNNG3LDH, which indicate higher rate of formation of ^•^OH radicals. Specifically, ^•^OH radical could be directly produced by the reaction of ^•^OH/OH^−^ with the photogenerated hole on semiconductor surface with an oxidation potential greater than +1.99 eV *vs*. NHE. Secondly, ^•^OH could be formed as an intermediate product during the reaction of ^•^O_2_^−^ radicals with H^+^ to produce hydroperoxy radical E^Ѳ^ (O_2_/^•^HO_2_ = −0.05 eV *vs*. NHE), which subsequently decomposed to ^•^OH active species^54^. The photogenerated holes on NiFe LDH (+2.19 eV *vs*. NHE) have sufficient oxidation potential to react with OH^−^/H_2_O to produce ^•^OH radicals. The photogenerated electrons on the CB edge of CN (−0.54 V *vs*. NHE) have sufficient potential to produce ^•^O_2_^−^ radicals, which further decomposed to ^•^OH active species^54^. In sum of, ^•^O_2_^−^ and ^•^OH radicals were produced over the CN surface and majority of ^•^OH radicals were produced on NiFe LDH surface. This charge separation could be attributed to the Z-scheme charge transfer mechanism with recombination of electrons and hole pairs over N-rGO surface (Fig. 16c,d). Based on ESR and TA-PL results, the photodegradation and corresponding mineralization of RhB and phenol over CNNG3LDH was justifiably follows Z-scheme mechanistic pathway.

## Conclusions

In summary, heterostructure NiFe LDH/N-rGO/g-C_3_N_4_ nanocomposite were successfully fabricated by combining calcinations-electrostatic self-assembly and hydrothermal steps. The outstanding photocatalytic performance of the heterostructure CNNG3LDH is mainly associated with the strong electrostatic bonding effect between NiFe LDH, N-rGO and g-C_3_N_4_ with effective charge separation by N-rGO mediator based Z-scheme mechanism.The morphology of CNNG3LDH as depicted from HRTEM images clearly reveals the intermixing of N-rGO with NiFe LDH/g-C_3_N_4_ hybrid. The ^•^OH and ^•^O_2_^−^ reactive species produced over the surface of CN and direct h^+^ oxidation to ^•^OH over the surface of NiFe LDH strongly proved the Z-scheme mechanistic path of CNNG3LDH and play major roles in the mineralization of RhB and phenol. Moreover, CNNG3LDH exhibit H_2_ production rate of 2508 μmolg^−1^ 2 h^−1^, which was enhanced by 1.68, 3.96 and 54.5 fold times than CNLDH, NiFe LDH and CN, respectively. In addition, CNNG3LDH exhibit O_2_ production rate of 1280 μmolg^−1^ 2 h^−1^, which was enhanced by 1.44, 3.91 and 64 fold times than CNLDH, NiFe LDH and CN, respectively. Overall, heterostructure CNNG3LDH possess realistic stability that enhances their superior photocatalytic performances and recyclability, thus making it a promising solar-light harvesting photocatalytic material as applicable in environmental mitigation and clean energy production sector.

## Experimental Section

### Chemicals

Graphite powder (Aldrich, 99%), Melamine (Aldrich, 99.0%), Ni(NO_3_)_2_. 6H_2_O (Aldrich, 99.0%), Fe (NO_3_)_3_. 9H_2_O (Aldrich, 99.0%), NaOH (Merck India, 99.5%), CH_3_OH (Merck India, 99%) and AgNO_3_ (Merck India, 99.9%), 4-aminoantipyrene (Merck India, 98%), potassium ferricyanide (Aldrich, 99.5%) were used as received. All other chemicals and solvents procured were of analytically pure grade and used as directly for the research work.

A detailed discussion on the synthesis of graphene oxide (GO) and g-C_3_N_4_/NiFe LDH (CNLDH) composite were provided in the experimental section of the supporting information.

### Preparation of NiFe LDH

Pristine NiFe LDH was synthesized according to our previously reported method with slight modification^5^. In a typical experiment, 0.02 M Ni (NO_3_)_2_. 6H_2_O (5.8162 g) and 0.004 M Fe (NO_3_)_3_. 9H_2_O (1.616 g) of metal salt (Ni^2+^: Fe^3+^ =5:1) were dispersed in 20 mL of deionized water and sonicated for 30 mins until a clear solution was obtained. To this suspension, 5.0727 g of NaOH in 10 mL of distilled water was added drop wise till the solution pH reached to 9 and then the gel was stirred for 12 h. The solid products obtained were collected by filtration, washed several times with distilled water followed by final rinse with ethanol three times each, and dried in vacuum at 60 °C for 24 h. For the preparation of layered NiFe LDH nanosheets, liquid phase exfoliation method was followed in which the brownish yellow coloured gel of NiFe LDH (approximately 2 gm) obtained after addition of NaOH solution were dispersed in formamide solvent (100 mL) for 30 mins. Then the resultant dispersed gel suspension was transferred into a Teflon lines stainless steel autoclave for hydrothermal treatment at 160 °C for 8 h. Subsequently, the product was centrifuged at 5000 rpm for 15 min to remove the remaining unexfoliated bulk materials. The collected supernatant possessed a dense concentration of about 20 mg mL^−1^.

### Preparation of g-C_3_N_4_

Pristine g-C_3_N_4_ was prepared by heating an appropriate amount of melamine at 550 °C for 2 h under N_2_ atmosphere according to our previously reported method^5^. The as-synthesized yellow coloured g-C_3_N_4_ was grounded to fine powder to get the desired pristine g-C_3_N_4_ designated as CN. The CN nanosheets were prepared by slight modification of bulk preparation method. In a typical procedure, 2 g of melamine were dissolved in 10 mL of double distilled water and were heated at 550 ^0^C for 2 h under protection of N_2_ atmosphere. Then 0.1 g of CN powder was sonicated in 10 mL of double distilled water to get an aqueous suspension of CN nanosheets.

### Preparation of N-GO/g-C_3_N_4_ hybrid

N-GO/g-C_3_N_4_ hybrid was prepared using GO and melamine by thermal annealing method. In a typical experiment, N-GO/g-C_3_N_4_ (x) nanohybrid, where x = 1, 3, 5 and 7 wt% of GO w.r.t the amount of NiFe LDH (1 g) was synthesized by mixing a calculated amount of melamine (0.03, 0.07, 0.11, 0.15 g) to an appropriate amount of GO (0.01, 0.03, 0.05 and 0.07 g) dispersion in 25 mL of double distilled water with vigorous stirring. According to the electrostatic interactions, the melamine would deposit on the GO surface during vigorously stirring at room temperature and then constantly stirred at 80 °C until dried. For complete drying it was further kept in an oven at 70 °C for overnight. The obtained GO/melamine products were grounded into powder and placed in a alumina crucible with a half cover lid inside the furnace, which was annealed at 550 °C for 2 h with a heating rate of 5 °C/min under flow of N_2._ The obtained series of products were N-GO/g-C_3_N_4_(x), which were denoted as CNNGx, where x = 1, 3, 5 and 7 wt% of GO w.r.t the amount of NiFe LDH (1 g).

### Preparation of heterostructure NiFe LDH/N-rGO/g-C_3_N_4_ nanocomposite

The obtained CNNGx products were dispersed in 20 mL of double distilled water to get an aqueous suspension of CNNGx nanosheets and then the suspension were immerged into the layered NiFe LDH nanosheets dispersion in formamide (1 g/50 mL) for 1 h to reach an electrostatic self-assembly equilibrium of the layered nanosheets. Then the whole dispersion was transferred into a 100 mL Teflon lined stainless steel autoclave for hydrothermal treatment at 160 °C for 8 h. The autoclave was cooled down naturally at room temperature to obtain the heterostructure NiFe LDH/N-rGO/g-C_3_N_4_ nanocomposite. Finally, the products were separated by centrifugation (8190 rpm for 15 min) and washed 3–4 times by using ethanol and dried at 80 °C for 24 h. Accordingly heterostructure NiFe LDH/N-rGO/g-C_3_N_4_ nanocomposite with x = 1, 3, 5 and 7 wt% of GO with respect to NiFe LDH in CNNGx were synthesized and named as CNNGxLDH. The heterostructure CNNGxLDH nanocomposite were compared with pristine NiFe LDH, pristine CN and CNLDH composites, respectively. Figure 1 elucidates the synthetic steps involved in heterostructure CNNGxLDH nanocomposite.

### Materials Characterization

The X-ray diffraction (XRD) measurements were performed on a PANanalytical X-ray diffractometer using a Cu-Kα source (I = 1.54 Å, 40 KV, 100 mA). The Fourier transform infrared (FTIR) spectra of the materials were measured on a Bruker Alpha FTIR spectrophotometer using KBr as reference for the measurement process. The diffuse reflectance UV (DRUV)-Vis spectra of the samples were measured using JASCO-V-750 UV-vis spectrophotometer connected with diffuse reflectance accessory in between the range of 200–800 nm using reference as pellets of boric acid. The photoluminescence (PL) spectra of the samples were measured on JASCO-FP-8300 Fluorescence spectrophotometer and excited at 380 nm. Time resolved photoluminescence (TRPL) spectra were measured at room temperature using a Fluoromax-4 spectrofluorometer (Horiba Scientific). The high resolution transmission electron microscopy (HRTEM), energy dispersive X-ray (EDX) were obtained on FEI, TECNAI G220, TWIN, Philips system with accelerating voltage of 200 kV. Atomic force microscopy (AFM) imaging was performed using a Nanoscope V system (Veeco). Zeta potentials were precised using a Malvern Zetasizer S. Total organic carbon (TOC) of the dye degraded liquid was analyzed by using Elementar-Vario TOC machine. Electron spin resonance (ESR) signals of spin-trapped paramagnetic species with 5, 5-dimethyl-l-pyrroline N-oxide (DMPO) were recorded with a Bruker A300E spectrometer with 100 kHz field modulation. The X-Ray photoelectron spectroscopy (XPS) measurements were performed on a VG Microtech Multi lab ESCA3000 spectrometer with a non-monochromatised Mg-Ka X-ray source and energy of 0.8 eV. The binding energy correction was performed by the C1s reference peak of carbon atom at 284.9 eV. The Raman spectra were measured with a Renishaw Raman microscope. The photoelectrochemical studies were performed by preparing working electrode of the as synthesized materials (NiFe LDH, CN, CNLDH and CNNG3LDH) through electrophoretic deposition method over coating the surface of fluorine-doped tin oxide (FTO). The as synthesized catalysts (~30 mg) were mixed with iodine powder (~20 mg) and acetone solution (30 mL), which then sonicated for 15 mins. Afterwards, two FTO electrodes facing parallel to each other were dipped into the solution and the separation between them is 10–20 mm. An applied potential of 60 V was fixed through potentiostat for about 3 minutes. The uniformity of the coated area of electrode fixed at 1 cm × 1 cm and finally dried at 80 °C for 2 h. The photoelectrochemical studies were performed on IVIUMn STAT electrochemical workstation. The component of electrochemical workstation consists of an aqueous solution of 0.1 M Na_2_SO_4_ as electrolyte with standard three-electrode cell attached with quartz pane and potentiostat–galvanostat. The FTO coated films, Pt and Ag/AgCl were used as the working electrode, the counter electrode and the reference electrodes, respectively. A 300 W Xe lamp (ORIEL) was used as the source for visible light. The electrochemical impedance (EIS) or Nyquist plot was measured at 10^5^ Hz to 1 Hz at a potential of 0.1 V in 0.1 M Na_2_SO_4_ solution by the presence of light in an open circuit potential. The Mott-Schottky (M-S) measurement was carried out at a constant frequency of 500 Hz under dark. The linear sweep voltammetry (LSV) plots were evaluated by a potential biasing of -1 to +1 V at a scan rate of 0.05 mV s^−1^ under both dark and light illuminations, respectively.

### Dark adsorption experiment of dye

The RhB adsorption experiment was conducted in a batch equilibration technique by pouring 20 mL of 20 mg/L of RhB dye (20 ppm) solution into 100 mL stopperd conical Pyrex flask. An amount of 20 mg of the as synthesized samples was added into the test solution at room temperature. For each experiment, the agitation time was fixed for 30 min. After agitation, the solution was centrifuged and the RhB concentration was measured in a JASCO 750 UV-Vis spectrophotometer at λmax of 554 nm. The equilibrium adsorption capacity and the RhB removal efficiency of the as prepared samples were estimated by using the following Eqs (7, 8).

7 $${\rm{Adsorption}}\,( \% )=\frac{{{\rm{C}}}_{0}-{{\rm{C}}}_{{\rm{e}}}}{{{\rm{C}}}_{0}}\times 100 \% $$

8 $${q}_{e}=\frac{{{\rm{C}}}_{0}-{{\rm{C}}}_{{\rm{e}}}}{{\rm{m}}}\times {\rm{V}}$$

where, C_e_ and C_0_ are the equilibrium concentrations (mg/L) of RhB at time t and C_0_ is the initial concentration at time t = 0 min, respectively. q_e_ is the adsorption capacity (mg/g), m and V are the mass (mg) of the as prepared samples and volume (L) of the solution, respectively.

### Photocatalytic experiment

#### Photodegradation experiment of organic pollutants (RhB and Phenol)

The photocatalytic degradation process was carried out by using 20 mL of RhB (20 ppm) solution consisting of 0.02 g of catalyst in a 100 mL stopperd conical Pyrex flask. The solutions were exposed to an average sun light intensity of 102,000 Lux. The solutions were stirred at a speed of 200 rpm for 120 min. The residual concentration of RhB was measured by a JASCO 750 UV-Vis spectrophotometer at λmax of 554 nm. The active species involved in the degradation process were detected by using various trapping agents such as p-benzoquinone (p-BQ), DMSO, IPA and EDTA acted as the scavengers for superoxide radical, electron, hydroxyl radical and hole, respectively. The reaction conditions were similar to that of degradation process but in addition 5 mM of trapping agents were added to the pollutant solution. Furthermore, the photo degradation experiments were repeated five times in order to study the reusability of efficient photocatalyst. After each cycle, the photocatalyst was washed with deionized water and ethanol, consecutively and dried in an oven. The dried samples were further used for another repetitive cycle. Similarly, the photocatalytic degradation of phenol was carried out by using 20 mL of phenol (20 ppm) containing 0.02 g of catalyst in 100 mL stoppered Pyrex conical flask. The experiments were carried out for 2 h in presence of sun light. The solutions were stirred at a speed of 200 rpm for 120 min. The residual concentration of phenol was measured by a JASCO 750 UV-Vis spectrophotometer at λmax of 510 nm. The spectroscopic analysis of the phenol degradation was carried out by developing the transparent golden colour. At first, 2 mL of 1 M NH_4_Cl solutions followed by conc. NH_4_OH solution was added to the phenol solution in order to maintain the pH of the solution in between 9.8–10.2. Afterwards, stoichiometric amounts of 4-aminoantipyrene and potassium ferricyanide were added to the solution to develop the persistent golden colour.

#### Water splitting experiment (H_2_ and O_2_ production)

Photocatalytic H_2_ production by water splitting reaction was carried out in a reactor attached with 125 W medium pressure visible light Hg lamps with 1 M NaNO_2_ as UV cut off filter to irradiate light of wavelength ≥400 nm. A power density of 100 mW cm^−2^ was measured for the visible light incident on the reaction chamber. The water splitting reactions were carried out by dispersing 0.03 g each of NiFe LDH, CN, CNLDH and heterostructure CNNGxLDH, x = 1, 3, 5 and 7wt % catalyst in an aqueous solution of 10 vol% of 30 mL CH_3_OH solution as sacrificial agent for H_2_ gas evolution. The catalyst suspensions were stirred constantly in order to prevent the settling down of the catalyst. The reactor was purged with N_2_ gas continuously for proper evacuation of the system before starting the experiment. The evolved H_2_ gas was detected by GC-17A and the column of which were packed with 5 Å molecular sieve equipped with thermal conductivity detector (TCD). Similarly, for O_2_ evolution reactions, the conditions were same as those maintained for H_2_ gas evolution with 0.03 g of catalyst mixed with an aqueous solution of 10 vol% of AgNO_3_ (30 mL) as sacrificial agent.

The apparent conversion efficiency (ACE) of the current photocatalytic system i.e. CNNG3LDH (H_2_ evolution yield 1300 μmol/h and O_2_ evolution yield 649 μmol/h by using 125 W Hg lamp as the visible light source positioned 9 cm away from the photocatalytic reactor) could be determined by using following Eq. (9):

9 $$\begin{array}{c}{\rm{ACE}}\,( \% )=\frac{{\rm{Stored}}\,{\rm{chemical}}\,{\rm{energy}}}{{\rm{Incident}}\,{\rm{light}}\,{\rm{energy}}}\times 100\\ {{\rm{H}}}_{2}{\rm{O}}\to {H}_{2}+\frac{1\,}{2\,}{{\rm{O}}}_{2}\,{\rm{\Delta }}{H}_{{\rm{c}}}=285.8\,{\rm{kJ}}/{\rm{mol}}\end{array}$$

where ΔH_c_ = heat of combustion of hydrogen in kJ/mol,

Stored chemical energy = (number of moles of hydrogen produced per second after the reaction) × ΔH_c_ = 0.1805 µmol/s × 285.8 kJ/mol = 0.0515 J/s or W.

The calculated power density for the visible light incident on the reaction chamber is approximately 100 mW cm^−2^. Therefore, incident light energy = 100 mW cm^−2^× (area of the spherical surface of the reaction chamber) = 100 mW cm^−2^× π × r^2^ (r is the radius of the circle and the value is measured to be 1.5 cm) = 100 mW cm^−2^ × 3.141 × (1.5 cm)^2^ = 0.7067 W.

Therefore, conversion efficiency (H_2_ evolution) = 0.0515 W/0.7067 W = 0.0729 = 7.29% Similarly, the conversion efficiency for O_2_ evolution reaction was calculated to be 3.64%

## Supplementary information

Supplementary Info
