# Supplementary material for: Deciphering Z-scheme Charge Transfer Dynamics in Heterostructure NiFe-LDH/N-rGO/g-C3N4 Nanocomposite for Photocatalytic Pollutant Removal and Water Splitting Reactions
Source: Sci Rep. 2019 Feb 21;9:2458. doi: 10.1038/s41598-019-39009-4 (PMC6385283; doi:10.1038/s41598-019-39009-4)
Supplement: Supplementary file 1 — Supplementary Info [file 41598_2019_39009_MOESM1_ESM.docx]

**Supporting Information**

**Deciphering Z-scheme Charge Transfer Dynamics in Heterostructure NiFe-LDH/N-rGO/g-C_3_N_4_ Nanocomposite for Photocatalytic Pollutant Removal and Water Splitting Reactions**

Susanginee Nayak ^a^ and K. M. Parida ^a^*

^a^ Centre for Nano Science and Nano Technology, Siksha ‘O’ Anusandhan University, Bhubaneswar-751030, Odisha, India

................................................................

^*^Corresponding author

*K.M. Parida*

*E-mail: kulamaniparida@soauniversity.ac.in & paridakulamani@yahoo.com*

*Tel. No: +91-674-2351777; Fax: +91-674-2350642*

**Experimental Section**

**Preparation of Graphene oxide**.

Graphene oxide (GO) was synthesized by using graphite powder using Hummers’ method. Typically, graphite powders (2 g) and NaNO_3_ (1 g) were mixed together and added into concentrated H_2_SO_4_ (46 mL, 98%) solution in an ice bath. KMnO_4_ (6 g) was gradually added into the mixture with constantly stirring to keep the temperature below 20 °C. After that, the ice bath was removed and the mixture was stirred at 35 °C in a water bath for 1 h followed by addition of H_2_O (90 mL). The color of the solution turned to yellowish with stirring at 98 °C for 1 h. Then the solution was further diluted with addition of distilled water (300 mL) followed by H_2_O_2_ (30%, 30 mL). After being kept settled for overnight, the remaining product was centrifuged and washed with distilled water until the pH became neutral. The product was dried in a vacuum to obtain the brown flakes of GO.

**Preparation of g-C_3_N_4_/NiFe LDH composite.**

In a typical preparation of g-C_3_N_4_/NiFe-LDH composites, an appropriate amount of pristine g-C_3_N_4_ was added into methanol solution and then ultrasonicated for 30 min to completely disperse the g-C_3_N_4_. Then the bulk NiFe LDH powder was dispersed for 30 min in methanol solution to get a complete dispersion of NiFe LDH. Afterwards, both the dispersed solutions of g-C_3_N_4_ and NiFe-LDH were mixed with each other and stirred in a fume hood for 24 h. After volatilization of the methanol, an opaque powder was obtained. The powder was dried at 100 ^0^C and again ground for further use. The obtained g-C_3_N_4_/NiFe LDH composite was denoted as CNLDH.

**Table S1.** Photogenerated charge lifetimes of NiFe LDH, CN, CNLDH and CNNGxLDH.

| **Catalyst** | **A1** | **τ1(ns)** | **A2** | **τ2 (ns)** | **τ_av_ (ns)** |
| --- | --- | --- | --- | --- | --- |
| NiFe LDH | 37% | 3.1 | 64% | 6.24 | 5.5 |
| CN | 58% | 5.9 | 47% | 3.5 | 5.1 |
| CNLDH | 13% | 10.2 | 10% | 5.1 | 8.6 |
| CNNG3LDH | 7% | 17.36 | 43% | 15.5 | 16.52 |

**Table S2.** K_app_ and Regression co-efficient (R^2^) values of RhB dye degradation over NiFe LDH, CN, CNLDH and CNNGxLDH.

| **Catalyst** | **RhB (K_app_ min^-1^)** | **Regression Coefficient (R^2^)** |
| --- | --- | --- |
| NiFe LDH | 0.0020 | 0.7910 |
| CN | 0.0074 | 0.9626 |
| N-rGO | 0.0083 | 0.9639 |
| CNLDH | 0.0104 | 0.9736 |
| CNNG1LDH | 0.0135 | 0.9694 |
| CNNG3LDH | 0.0174 | 0.9929 |
| CNNG5LDH | 0.0161 | 0.9875 |
| CNNG7LDH | 0.0054 | 0.9667 |

**Table S3.** K_app_ and Regression co-efficient (R^2^) values of phenol degradation over NiFe LDH, CN, CNLDH and CNNGxLDH

| **Catalyst** | **Phenol (K_app_ min^-1^)** | **Regression Coefficient (R^2^)** |
| --- | --- | --- |
| NiFe LDH | 0.0046 | 0.9477 |
| CN | 0.0069 | 0.9393 |
| N-rGO | 0.0071 | 0.9681 |
| CNLDH | 0.0074 | 0.9515 |
| CNNG1LDH | 0.0083 | 0.9528 |
| CNNG3LDH | 0.0083 | 0.9707 |
| CNNG5LDH | 0.0080 | 0.9070 |
| CNNG7LDH | 0.00509 | 0.9573 |

**Table S4.** Comparison results of photocatalytic RhB dye degradation activities of CNNG3LDH with other reported material.

| **Catalytic system** | **Concentration of RhB** | **Catalyst dose** | **Degradation rate (%)** | **Time study** | **Source** | **Reference** |
| --- | --- | --- | --- | --- | --- | --- |
| CNNG3LDH | 20 mg/L | 0.02 g | 97 | 2 h | Sun Light | Present work |
| ZnCr-LDH/graphene | 10 mg/L | 0.1 g | 88 | 140 min | 300 W Xe Lamp | Ind. Eng. Chem. Res. **53**,12943–12952 (2014) |
| BiVO_4_/g-C_3_N_4_ | 1×10^-5^ mol/L | 0.05 g | 85 | 5 h | 500 W Xe Lamp | *Dalton Trans.* **44**, 4297-4307 (2015) |
| AgCl/Ag/ γ TaON | 1×10^-4^ mol/L | 0.1 g | 96.6 | 140 min | 300 W Xe Lamp | *Appl. Catal. B,* **579**, 142–143 (2013) |
| Bi_2_O_3_/g-C_3_N_4_ | 1×10^-5^ mol/L | 1 g | 45.6 | 60 mins | 500 W Xe arc Lamp | *J. Hazard. Mater*, **280**, 713-722 (2014) |

**Table S5.** Comparison results of phenol photocatalytic degradation activities of CNNG3LDH with other reported material.

| **Catalytic system** | **Concentration of Phenol** | **Catalyst dose** | **Degradation rate (%)** | **Time study** | **Source** | **Reference** |
| --- | --- | --- | --- | --- | --- | --- |
| CNNG3LDH | 20 mg/L | 0.02 g | 75 | 2 h | Sun Light | Present work |
| CeO_2_/Mg-Al LDH | 0.85 mmol/L^-1^ | 0.2 g | 50 | 7h | UV light using a Pen Ray Power Supply lamp, 4400µW cm^−2^ | *Applied Catalysis B: Environmental,***102**, 276–285 (2011) |
| TiO_2_/Graphene | 50 mg/L | 0.05 g | 68 | 360 min | Visible Light | *RSC Adv.,* **6**, 96554-96562 (2016) |
| 1.2% WO_3_@RGO | 10 mg/L | 0.01g | 30 | 240 min | Simulated solar Light | *Appl. Catal., A*, **522**, 90-100 (2016) |
| Pt/RGO | 0.5 mM | 0.5 g/L | 51.6 | 180 min | Solar Light | *Nanoscale*, **8**, 19174- 19175 (2016) |
| 5%RGO/Fe_2_O_3_ | 10 mg/L | 0.01 g | 67 | 120 mins | Visible Light | *ACS Sustainable Chem. Eng.,* **5**, 10551- 10562(2017) |

**Table S6.** Comparison results of photocatalytic H_2_ production activities of heterostructure CNNG3LDH with other reported material.

| **Catalytic system** | **Source** | **H_2_ Production** | **Reference** |
| --- | --- | --- | --- |
| CNNG3LDH | Hg Lamp (125 W) | 1300 (µmol g^-1^h^-1^) | Present work |
| Au/ZnCeAl-LDH | Solar Simulator (1300 W) | 127 (µmol g^-1^h^-1^) | ***J. Mater. Chem. A***, **1**, 9092-9098(2013) |
| CdS/ZnCr-LDH | Xe Lamp (300 W) | 374 (µmol g^-1^ h^-1^) | *RSC Adv.* **5**, 5823-5829(2015) |
| CdS/Cd/ZnO | Xe lamp (300 W) | 1920 (μmol g^–1^h^–1^) | *Adv. Energy Mater.*, **2**, 42-46 (2012) |
| CdS/Au/TiO_2_ | Xe lamp (750 W) | 64 (μmol g^–1^h^–1^) | *Int. J. Hydrogen Energy*, **38**, 8244-8253(2013) |
| Pt/CdS/Au/TiO_1.96_ C_0.04_ | Xe lamp (300 W) | 433.2 (μmol g^–1^h^–1^) | *ACS Nano,* **5**, 4084-4090(2011) |
| CuGaS_2_/RGO/TiO_2_ | Xe lamp (300 W) | 19.8 (μmol g^–1^h^–1^) | *J. Am. Chem. Soc.* **137**, 604-607 (2015) |
| Si/TiO_2_ | Xe Lamp (300 W) | 875 (µmol g^-1^ h^-1^) | *Nano Lett.* **13**, 2989-2992 (2013) |
| Pt/TiO_2_- anatase/TiO_2_-rutile | Hg lamp (400 W) | 180 (μmol g^–1^h^–1^) | *Chem. Phys. Lett.* **344**, 339-343(2001) |

**Table S7.** Comparison results of photocatalytic O_2_ production activities of CNNG3LDH with other reported material.

| **Catalytic system** | **Source** | **O_2_ Production** | **Reference** |
| --- | --- | --- | --- |
| CNNG3LDH | Hg Lamp (125 W) | 649 (µmol g^-1^h^-1^) | Present work |
| PbBi_2_Nb_1.9_ Ti_0.109_/W/WO_3_ | Xe Lamp (450 W) | 741 (µmol g^-1^h^-1^) | *Appl. Phys. Lett.* **89**,064103-064106(2006) |
| Si/TiO_2_ | Xe Lamp (300 W) | 458 (µmol g^-1^ h^-1^) | *Nano Lett.* **13**, 2989-2992(2006) |
| CuGaS_2_/RGO/TiO_2_ | Xe lamp (300 W) | 10.2 (μmol g^–1^h^–1^) | *J. Am. Chem. Soc.* **137**, 604-607(2015) |
| Ru/SrTiO_3_:Rh/RGO/BiVO_4_ | Xe lamp (300 W) | 5.5 (μmol g^–1^h^–1^) | *J. Am. Chem. Soc.,* **133***,* 11054-11057(2011) |
| CdS/Ti_0.91_O_2_ | Xe lamp (300 W) | 0.18 (μmol g^–1^h^–1^) | *Chem.Commun.* **51**, 13354-13357(2015) |
| Pt/TiO_2_-anatase/TiO_2_-rutile | Hg lamp (400 W) | 90 (μmol g^–1^h^–1^) | *Chem. Phys. Lett.* **344**, 339-343(2001) |

**Figure S1.** (a) FTIR spectra of NiFe LDH, CN, CNLDH and CNNG3LDH, (b) Raman spectra of NiFe LDH, CNLDH and CNNG3LDH and (c) Zeta Potential study of CNLDH and CNNG3LDH.

**Figure S2.** TEM image of (a) CN, (b) N-rGO, (c) CNNG and HR-TEM image of (d) NiFe LDH.

**Figure S3.** (a) A layered image and SEM elemental mapping of CNNG3LDH demonstrating uniform presence of (b) Ni, (c) Fe, (d) C, (e) O, and (f) N.

**Figure S4.** Adsorption *vs*. degradation evaluation of NiFe LDH, CN, N-rGO, CNLDH and CNNGxLDH towards RhB degradation (conditions: catalyst dose=0.02 g, [RhB] = 20 ppm, dark light= 30 min, solar light exposure time=120 min).

**Figure S5.** (a) Effect of various scavengers on the photodegradation of RhB over CNNG3LDH, (b) ESR spectra of NiFe LDH, CN and CNNG3LDH, and (c) TAOH fluorescence spectral emission behaviour of NiFe LDH, CN, CNLDH and CNNG3LDH.

**Figure S6.** TOC study of RhB (20 ppm) and phenol (20 ppm) over CNNG3LDH.

**Figure S7.** XRD spectra of CNNG3LDH before and after H_2_ evolution and RhB degradation reaction.

**Figure S8.** XPS spectra : (a) Ni 2p, (b) Fe 2p, (c) N 1s, (d) O 1s, and (e) C1s of CNNG3LDH after H_2_ evolution and RhB degradation reaction
